# The Role of PKM2 in Metabolic Reprogramming: Insights into the Regulatory Roles of Non-Coding RNAs

**DOI:** 10.3390/ijms22031171

**Published:** 2021-01-25

**Authors:** Dexter L. Puckett, Mohammed Alquraishi, Winyoo Chowanadisai, Ahmed Bettaieb

**Affiliations:** 1 Department of Nutrition, University of Tennessee Knoxville, Knoxville, TN 37996, USA; dpucket4@vols.utk.edu (D.L.P.); Malqurai@vols.utk.edu (M.A.); 2 Department of Nutrition, Oklahoma State University, Stillwater, OK 74078, USA; winyoo.chowanadisai@okstate.edu

**Keywords:** pyruvate kinases, cancer metabolism, metabolic reprogramming, long non-coding RNAs

## Abstract

Pyruvate kinase is a key regulator in glycolysis through the conversion of phosphoenolpyruvate (PEP) into pyruvate. Pyruvate kinase exists in various isoforms that can exhibit diverse biological functions and outcomes. The pyruvate kinase isoenzyme type M2 (PKM2) controls cell progression and survival through the regulation of key signaling pathways. In cancer cells, the dimer form of PKM2 predominates and plays an integral role in cancer metabolism. This predominance of the inactive dimeric form promotes the accumulation of phosphometabolites, allowing cancer cells to engage in high levels of synthetic processing to enhance their proliferative capacity. PKM2 has been recognized for its role in regulating gene expression and transcription factors critical for health and disease. This role enables PKM2 to exert profound regulatory effects that promote cancer cell metabolism, proliferation, and migration. In addition to its role in cancer, PKM2 regulates aspects essential to cellular homeostasis in non-cancer tissues and, in some cases, promotes tissue-specific pathways in health and diseases. In pursuit of understanding the diverse tissue-specific roles of PKM2, investigations targeting tissues such as the kidney, liver, adipose, and pancreas have been conducted. Findings from these studies enhance our understanding of PKM2 functions in various diseases beyond cancer. Therefore, there is substantial interest in PKM2 modulation as a potential therapeutic target for the treatment of multiple conditions. Indeed, a vast plethora of research has focused on identifying therapeutic strategies for targeting PKM2. Recently, targeting PKM2 through its regulatory microRNAs, long non-coding RNAs (lncRNAs), and circular RNAs (circRNAs) has gathered increasing interest. Thus, the goal of this review is to highlight recent advancements in PKM2 research, with a focus on PKM2 regulatory microRNAs and lncRNAs and their subsequent physiological significance.

## 1. Introduction

Globally, and in the U.S., cancer remains a leading cause of death and continues to pose one of the most substantial burdens to humanity’s health and wellbeing [1]. Advances in cancer research are constantly pushing the boundaries of our understanding of how the nature of cancer metabolism may be exploited in order to establish improved therapeutic strategies. The identified phenomena have revealed mechanisms through which cancer cells can augment and rewire nutrient metabolism to support their accelerated growth requirements. Indeed, cancer cells are capable of increasing the uptake and extracellular influx of nutrients, partially through upregulating the expression of glucose [2] and amino acid transporters [3]. In some cases, cancer cells adopt mechanisms to acquire proteins from the extracellular fluid and subsequently use them as a pool to synthesize free amino acids [4]. In part, this mechanism enables cancer cells to obtain sufficient glutamine [5] to prompt nucleotide synthesis [6]. Importantly, cancer cells could alter intracellular metabolism to favor anabolic pathways, such as the shift in metabolism observed between oxidative phosphorylation and glycolysis [7]. This shift toward glycolysis has been speculated to be driven by increased expression of multiple genes, including HIF1α [8], c-Myc [9], and mTOR signaling [10]. Despite this, glycolysis is considered energetically inefficient compared to oxidative phosphorylation, with only two ATP molecules produced. However, glycolysis has been shown to generate its ATP at a faster rate [11]. Consequently, the increase in glycolysis and reduction in oxidative phosphorylation results in the accumulation of glycolytic metabolites, which can be used as an intermediate for the biosynthesis of both lipids [12] and amino acids [13]. Furthermore, the decrease in oxidative phosphorylation promotes the buildup of tricarboxylic acid cycle (TCA cycle) metabolites such as citrate, which then can be utilized as a precursor for lipid synthesis through its conversion into acetyl-CoA [14]. Finally, it is worth noting that out of these metabolic alterations, increased glycolysis remains the main and most extensively studied hallmark of cancer. This unique metabolic transformation for producing energy was first observed by Otto Warburg in 1926 [15] and became widely known as the Warburg effect.

The Warburg effect promotes cancer cell survival and expansion through several proposed mechanisms [16,17,18]. Increased glucose uptake and production of lactate are integral to tumor proliferation and remain the hallmark of the Warburg effect [15,16,19]. Moreover, Crabtree demonstrated that cancer cells could adapt to specific environmental and genetic circumstances [20], allowing cancer cells to shift between respiration and fermentation, thus promoting their evasive nature [16,20]. However, the Warburg effect was not considered pathologically significant until its role in cancer cell metabolism became more apparent [21,22]. Congruently, these findings created a high level of interest within the scientific community and resulted in the emergence of the Warburg effect as a promising target for pharmacological intervention and treatment of cancer [16,19,23].

In order to develop efficient therapeutic strategies targeting oncogenic transformation, a complete understanding of cancer metabolism is required. Recent studies have identified the Warburg effect as an essential contributor to cell proliferation through several mechanisms including the modulation of cell cycle machinery [24,25]. For instance, the anaphase-promoting complex/cyclosome-Cdh1 (APC/C-Cdh1) pathway has been identified as a glycolytic and cell cycle regulator, and an extension of the Warburg effect [24]. Innovative approaches to targeting the biological complexity of oncogenesis continue to reveal new possibilities.

Glycolysis is composed of multiple reactions that control the glycolytic flux, such as the reaction catalyzed by pyruvate kinases (PK) to generate pyruvate and ATP [26,27]. Interestingly, cancer cells shift towards the preferential expression of a specific isoform of PK known as PK isoform M2 (PKM2) [28]. PKM2 benefits cancer cells by promoting their adaptability to varying environmental conditions and improving their chances for survival [28]. Additionally, the cellular expression of PKM2 results in increased lactate production even under aerobic conditions [15,29]. The conversion of pyruvate into lactate excludes mitochondrial respiration and would seem energetically unfavorable [30]. These differences in metabolism between normal and cancer cells emerged as a focal point of cancer research and led to the pursuit of novel approaches targeting PKM2 as a potential target for cancer therapy [28,29]. In this review, we explore the different PKM2 functions, post-translational modifications, molecular mechanisms of regulation, and its overall contribution to healthy and pathological conditions. In addition, we will discuss the emergence and outcomes of novel findings demonstrating the potential of microRNAs (miRNAs) and long non-coding RNAs (lncRNAs) as potent regulators of PKM2 expression and functions.

## 2. PKM2: Uncovering the Origin

### 2.1. PKM2 Transcription and Dynamic Regulation

Mammalian pyruvate kinase is expressed as one of four different isoforms (M1, M2, L, and R) encoded by two distinct genes (PKM and PKLR) [30]. These isoforms share similar features, where they catalyze the final step in glycolysis and exhibit the same primary structure containing four major domains: A, B, C, and N [31,32,33,34]. However, the PK isoforms differ in their enzymatic potential, allosteric regulation [35], amino acid sequence, tissue distribution [14,36], and contribution to health and disease [37,38]. PKM1 and PKM2 are both expressed from the *PKM* gene and conversed across vertebrates [39]. The amino acid sequence for PKM2 is highly similar between humans and mice at 82% similarity [40]. The PKM gene is located on chromosome 15 in humans and chromosome 9 in mice [41]. The human PKM gene has 12 exons and 11 introns [42]. The two PK transcript isoforms M1 and M2 result from alternative splicing regulated by several spliceosomes including the heterogeneous nuclear ribonucleoprotein A1 and A2 (hnRNPA1 and hnRNPA2) and polypyrimidine tract binding protein (PTB) [43,44]. The inclusion of exon 9 and exclusion of exon 10 produces PKM1, whereas PKM2 includes exon 10 but not exon 9 [42]. Moreover, recent studies have shown that the insertion of exon 10 into the final PKM2 RNA is promoted through the action of the serine/arginine-rich splicing factor 3 (SRSF3) [45]. Both exon 9 and exon 10 are 167 base pairs and 56 amino acids in length [46], and the human PKM1 and PKM2 isoforms are both 531 amino acids long [32]. Consequently, the resulting M1 and the M2 isoforms differ by 22 amino acids located between amino acids 389 and 433 of the C-terminus domain [32]. The other two PK isozymes, PKL and PKR, are encoded by the PKLR gene, which is on chromosome 1 in humans and distinct from the PKM gene [47]. The human PKL and PKR isozymes still share approximately 71–72% amino acid similarity with PKM1 and PKM2, despite being transcribed from different genes [47]. Alternative splicing produces the R isoform [48], a 574 amino acid long protein that is strictly expressed in erythrocytes, and the L isoform, a 543 amino acid long protein that is highly expressed in the liver [30] and other tissues [49,50].

Even though all PK isoforms perform a similar enzymatic function, these isoforms differ in their kinetic properties and affinity towards phosphoenolpyruvate (PEP), while their affinity potential toward ADP remains comparable [33]. PKM2 exhibits the lowest basal enzymatic activity [51] and is the only isoform, to our knowledge, capable of existing in the enzymatically active “R-State” or inactive tetramer “T-State”, dimer, and monomer configurations [52]. This enables PKM2 to substantially alter its dynamics by existing in either the dimeric (high Km for PEP) and tetrameric forms (low Km for PEP) [53] to meet differential metabolic demands. The equilibrium of PKM2 configurations is tightly regulated by allosteric effectors, altering PKM2 kinetics and Km values for PEP [54]. In contrast, PKM1 predominantly exists in an active tetrameric form [55]. Similarly, the unphosphorylated PKL is considered active with higher affinity for PEP (K_0.5_ = 0.3 mM) in comparison to the phosphorylated form (K_0.5_ = 0.8 mM) [56]. However, under abnormal conditions, PKR was reported to exist in a mutated form with a tendency to dissociate into dimeric or monomeric configurations with altered Km value compared to unmutated enzyme [57]. Furthermore, PKM2 exhibits lower V_max_ compared to PKM1 [52], even though the fructose-1,6-bisphosphate (FBP) binding pockets of M1 and M2 are almost identical. The only reported difference is the presence of a glutamate residue in the M1 isoform instead of lysine in the M2 isoform [58]. Although minor, this difference was demonstrated to play a significant role in blocking the allosteric regulation of FBP in PKM1; however, it does not fully explain the kinetic variation between PKM isoforms.

Notably, the different PK isoforms are expressed in a tissue-specific manner that seems to be dependent upon energy requirements and the availability of nutrients [26,59]. For instance, PKL plays a role in gluconeogenic organs such as the kidney, liver, and small intestine [26,60] and can be phosphorylated and inhibited in response to high cellular levels of glucagon and ATP [59]. On the other hand, PKM1 is highly abundant amongst differentiated tissues (heart, brain, muscle, stomach, bone, skin, among others) where energy is produced and used rapidly [59]. PKM2, however, is expressed in the embryonic stages initially and, in most cases, is gradually replaced by other PK isoforms [14]. Notably, it has been revealed that various differentiated tissues continue to express PKM2 across the lifespan [30,61]. PKM2 also differs from other PK isoforms through its ability to translocate to the nucleus and regulate the transcription of numerous genes with key functions in a plethora of cellular processes further discussed below [62]. Additionally, while other PK isoforms exist in a stable tetrameric configuration, PKM2 may switch between the dimer or tetramer form in response to biological circumstances and metabolic needs [28]. This unique property of PKM2 allows for dynamic metabolic regulation, due in part to the variation in the affinity of the dimer and tetramer configurations of PKM2 to PEP.

### 2.2. Impact of PKM2 Mutations on Gene Expression

PKM2 expression, subcellular localization, and activity are regulated by several mechanisms. At the gene level, earlier studies have identified two missense mutations of PKM2 (H391Y and K422R) that could support the aggressive nature of cancer metabolism [63]. The two mutations are both specific to PKM2 but not PKM1 since they are encoded by exon 10 and were discovered in Bloom syndrome cells (H391Y) and a Bloom syndrome patient (K422R) [64]. Iqbal et al. transfected H1299 cells with either mutant or wild-type PKM2 mimicking the missense mutations, H391Y and K422R, and demonstrated that these missense mutations promote cancer proliferation through a variety of proposed metabolic alterations [63]. Cells transfected with the mutant PKM2 exhibited higher glucose uptake and lactate production, concomitant with a reduction in oxidative stress [63]. Moreover, in recent studies by Chen and colleagues, mutations in the exon 10 region of the *PKM* gene have been proposed to promote the translocation of PKM2 to the nucleus and have been associated with increased activity of the hypoxia-inducible factor 1-alpha (HIF-1α) [65]. HIF-1α is a well-established oxygen sensor in tumor cells and also a modulator of glycolysis and PKM2 expression through direct regulation of the c-Myc/hnRNP splicing axis to favor PKM2 expression [61]. In another study by M.V. Vander Heiden’s group, the authors argued that since PKM2 is not required for the growth of several cancers, as demonstrated by earlier studies, loss-of-function mutations observed in some human tumors are not oncogenic but rather help to create a metabolic state that favors the proliferation of tumor cells [33]. Further efforts towards a comprehensive understanding of the metabolic and physiological consequences of PKM2 mutations as well as their associated clinical outcomes are needed.

As noted above, PKM2 is highly expressed during neonatal stages and phases of proliferation, a fact that may explain the increase in PKM2 expression in tumors given their highly proliferative nature and the associated metabolic requirements. For instance, the oncogenic transcription factor c-Myc enhances the expression of PKM2 through upregulating the expression of *PKM* spliceosomes [44]. Similarly, the activation of the rapamycin (RTK/PI3K/AKT/mTOR) signaling pathway in tumor suppressor (Tsc1/2) deficient mouse embryonic fibroblasts (MEF) leads to a cascade of events that upregulates the levels of HIF-1α and, subsequently, increased PKM2 levels. Comparable to c-Myc, mTOR activation can promote tumorigenesis and metabolic transformation [66] and was shown to be integral to the oncogenesis, and the transition towards the Warburg effect [61].

A large number of factors have been shown to modulate the quaternary structure and physical configuration of PKM2, thus altering its enzymatic activity and subcellular localization acids long [30,32]. For example, the cis-trans isomerization plays a critical role in mediating the non-enzymatic function of PKM2 [67,68,69] through its conversion from a tetramer to a dimer or monomer. Although the tetrameric form is considered the active form and a higher tetramer/dimer ratio results in a higher conversion rate of PEP to pyruvate [30,60], PKM2 in tumor cells exists predominantly in the dimeric form and has been directly correlated with increased levels of lactate. It is likely that the high levels of dimer PKM2 relate to the “damming up effect” or the accumulation of glycolytic phospho-metabolites [30]. Meanwhile, the cis-trans isomerization of PKM2 and its transition between the tetramer and dimer forms can drastically alter its localization and functions. In tumors, the altered configuration of PKM2 provides cancer cells with the excess amino acids, nucleotides, and phospholipids needed for biosynthetic pathways during proliferation [30]. Notably, post-translational modifications play a key role in regulating the cis-trans isomerization of PKM2 and the associated metabolic consequences. For example, serine phosphorylation of PKM2 at position 37 (Ser-37) by ERK1/2 facilitates the recruitment of peptidyl-prolyl cis-trans isomerase NIMA-interacting 1 (PIN1), which mediates PKM2 cis-trans isomerization [67,69]. This conformational change exposes the nuclear localization signal (NLS) and results in the translocation of PKM2 to the nucleus, a process that requires the binding of PKM2 to importin α5 [67].

In addition to the cis-trans regulation of PKM2, several other factors were demonstrated to alter the quaternary structure and physical configuration of PKM2 acids long [30,32], PKM2 subcellular localization, and, subsequently, its functions. For instance, it is well established that phenylalanine acts as an allosteric inhibitor for both PKM1 [70] and PKM2 [52], thus reducing their affinity to PEP. FBP, on the other hand, is an allosteric regulator that promotes PKM2 tetramerization [71], resulting in PKM2 activation and the subsequent increase in glucose utilization [72]. Unlike PKM2, PKM1 lacks the regulatory effect of FBP due to differences in the orientation of the FBP-activating loop [32], which results in a significant reduction in PKM1’s ability to sense glucose. Accordingly, PKM2 missense mutations could potentially alter glucose uptake in cancer cells [63]. Moreover, SAICAR (succinyl-5′-phosphoribosyl-5-amino-4-imidazole carboxamide) and serine have also been identified as independent stimulants of PKM2 activity [73,74]. SAICAR allosterically stimulates PKM2 in a nutrient-dependent manner [74], while serine acts as an allosteric activator and ligand of PKM2 and both may play a critical role in the metabolic transformation required in oncogenesis [73]. These allosteric regulators could aid cancer cells in the metabolic transformation, allowing them to thrive in an environment limited in nutrients [51].

Moreover, post-translational modifications of PKM2 through oxidation, phosphorylation, and acetylation can also modify its activity, conformation, and localization [51]. Phosphorylation of PKM2 at tyrosine 105 residue (Tyr-105) stabilizes the dimer configuration, leading to inactivation of PKM2’s glycolytic activity [27]. A similar reduction in glycolytic function was also seen in response to PKM2 oxidation at cysteine (Cys)-358 which results in the entrance of glucose into the pentose phosphate pathway [75]. PKM2 is sensitive to oxidation by several oxidants including nitric oxide (NO), endothelial NO synthase (eNOS), and hydrogen peroxide (H_2_O_2_), all of which were demonstrated to be capable of regulating PKM2’s activity and its subcellular localization [14]. Notably, the redox regulation of PKM2 was shown to have substantial effects on both cancerous and non-cancerous metabolic outcomes. Therefore, it is imperative to consider redox homeostasis when investigating PKM2, although more research is still needed for a better understanding of the clinical impact of the full scope of oxidants and their regulation of PKM2 in metabolic transformation. It is worth noting, however, that alterations in PKM2 activity through oxidation in tumors facilitate cancer cells’ adaptation to oxidative stress through multiple distinct pathways. Post-translational modifications that reduce PKM2 activity, such as the oxidation of Cys-358 [75] and the dessuccinylation of Lys-498 [76] residues, increase the accumulation of glycolytic metabolites that promote glucose entrance into the pentose phosphate pathway, which generates reduced equivalents in the form of NADPH to clear excessive oxidant accumulation and maintain cancer cell survival. In addition, recent studies have shown that the PKM2-specific Cys-424 plays a crucial role in its conformational change and the transition between the tetrameric and dimeric forms. Mutation of this residue to leucine resulted in a higher tetramer to dimer ratio and resistance to oxidative stress-induced oxidation and inhibition of PKM2 [77].

### 2.3. Regulation of PKM2 Subcellular Distribution

The functions of PKM2 and its location within the cells are heavily dependent on its final assembled structure [30]. In the cytosol, PKM2 exhibits both tetrameric and dimeric isoforms and mainly converts PEP to pyruvate and controls a key regulatory step in glycolysis [29]. However, within the nucleus, PKM2 exists in the dimeric form and is involved in the regulation of gene expression [62]. The nuclear translocation of PKM2 is shown to be dependent upon a variety of complex protein–protein interactions. Recently, it has been demonstrated that the phosphorylation of PKM2 at Ser-37 by extracellular signal-regulated kinase 2 (ERK2) could ultimately allow the proper conformational change required for PKM2 translocation into the nucleus [62], a process that requires the binding of PKM2 to importin α5 [62]. The nuclear accumulation of PKM2 promotes the phosphorylation of histone H3, which can promote mitotic chromatin condensation [78], and upregulates the transcription of cell-cycle-regulating genes including *MYC* and *CCND1* [62]. Additionally, nuclear PKM2 was shown to play a key role in breast cancer cell proliferation and angiogenesis through modulation of epidermal growth factor receptor (EGFR) signaling and its downstream miR-148a and miR-152 genes. Furthermore, evidence suggests a direct interaction between PKM2 and the p65 subunit of nuclear factor kappa light chain enhancer of activated B cells (NF-κB), a well-established factor involved in cancer development and progression [79]. Furthermore, the nuclear translocation of the dimeric form of PKM2 was shown to be responsible for mediating HIF-1α function in the transition towards aerobic glycolysis [80]. According to recent studies, the interaction between PKM2 and HIF-1α leading to the activation of the latter’s transcriptional activity is dependent upon PKM2 hydroxylation at proline residues 403 and 408 by prolyl hydroxylase 3 (PHD3) [81]. Importantly, this interaction between the two proteins underscores the role of PKM2 in several aspects of cancer biology, given the role of HIF-1α in tumor progression, angiogenesis, invasion, metastasis, as well as adaptation to oxidative stress caused by exposure to chemicals and radiation [82,83,84].

In the nucleus, PKM2 was also shown to play a critical role in regulating β-catenin expression and downstream signaling with profound effects on the cell cycle, survival, and proliferation of tumor cells. Increased β-catenin levels have been implemented as a potential contributor to cancer development and proliferation [62,85]. The precise mechanisms by which PKM2 interacts and regulates β-catenin have been described previously [62,86,87] and were suggested to be essential to cancer cell proliferation [62,87]. Yang et al. identified that EGFR-activated ERK phosphorylates PKM2 but not PKM1, promoting PKM2 binding to importin α5 and its subsequent nuclear translocation [67]. Within the nucleus, PKM2-mediated phosphorylation of β-catenin at Y333 results in the subsequent induction of c-Myc [62]. Supportively, in another study, the activation of EGFR signaling resulted in PKM2-dependent β-catenin phosphorylation at Y333 and subsequent upregulation of c-Myc expression [87]. Consistent with these findings, in a more recent study, PKM2 silencing reduced the nuclear accumulation of β-catenin [88]. Likewise, the downregulation of PKM2 expression in Hep3B cells suppressed β-catenin activity and promoted its proteolytic degradation [89]. Conversely, the overexpression of PKM2 negatively modulated β-catenin signaling through a mechanism that was proposed to be dependent on the upregulation of miR-200a [86]. Interestingly, in thyroid cancer (TC) cells, the interaction between PKM2 and β-catenin was recently demonstrated to be dependent upon AMPK activation [90]. In this study, the binding of AMPK to PKM2 promoted β-catenin nuclear translocation and was deemed necessary for the migration of TC cells. Notably, findings from this study suggest that PKM2/β-catenin interaction and perhaps phosphorylation occur in the nucleus as PKM2 deficiency suppressed the nuclear accumulation of β-catenin, but not AMPK. Regardless, when combined, these studies emphasize the importance of the regulatory actions that PKM2 can exert on the β-catenin pathway. Moreover, the evidence shows that the induced nuclear activity and translocation of PKM2 can result in diverse cellular and metabolic outcomes, warranting continued exploration beyond its known cytosolic functions.

Outside the nucleus, PKM2 has been detected within other subcellular fractions including the mitochondria [91,92] and exosomes [93,94]. Under increasing oxidative stress, PKM2 can translocate to the mitochondria, where it can inhibit apoptosis through the phosphorylation and stabilization of BCL2 [91]. Likewise, glucose deprivation can lead to PKM2 succinylation and its mitochondrial translocation in HCT116 cells. Subsequently, this translocation resulted in an increase in ATP generation and mitochondrial permeability through inhibiting voltage-dependent anion channel 3 (VDAC3) ubiquitination, promoting cancer cell survival [92]. Recent studies have also identified a novel mechanism through which PKM2 regulates cancer cells’ interaction with their microenvironment through exosome release. Indeed, Wei and colleagues demonstrated that PKM2 could enable the release of exosomes through the phosphorylation of synaptosome-associated protein 23 (SNAP-23) and subsequent formation of the SNARE complex [93]. Exosomes have been shown to play critical roles in tumorigenesis through their role in promoting growth and expansion [95]. Taken together, these findings emphasize the crucial role that PKM2 may exhibit as a key regulator of various aspects of tumorigenesis through its ability to modulate multiple signaling pathways at different subcellular locations.

## 3. “Metabolic” and “Non-Metabolic” Functions of PKM2

### 3.1. PKM2 Glycolytic Function

Integral to the glycolytic pathway, PK results in the conversion of PEP + ADP into pyruvate and ATP, which then enter into the TCA cycle and ultimately undergo oxidative phosphorylation [96]. However, as mentioned above, different PK isoforms can result in varying metabolic fates and cellular outcomes. For example, it is well known that PKM1 is expressed in tissues that display high oxidative phosphorylation and overall mitochondrial ATP production. Conversely, PKM2 expression directly correlates with increased lactate production within in vivo and in vitro experimental models [71]. Dimeric PKM2 is able to shunt the energy production generated by the TCA cycle, in part due to less availability of pyruvate and acetyl-CoA for oxidative phosphorylation [30,97]. As evidenced, PKM2 has also been identified in the mediation of various non-glycolytic roles and functions.

### 3.2. PKM2 Non-Glycolytic Functions

Recently, research investigating the various functions and interactions of PKM2 beyond glycolytic function has emerged [27,51,98,99]. Indeed, substantial evidence suggests that PKM2 plays a key role in the adaptation of tumor cells to oxidative stress. This is evident through its critical roles in the regulation of HIF-1α and its downstream target genes [44]. As previously mentioned, HIF-1α plays a regulatory role within cancer metabolism through its ability to shift cancer cells towards the Warburg effect [100]. In addition, HIF-1α acts as an activator for PKM2 transcription, potentially creating a reciprocal transcriptional regulatory loop between PKM2 and HIF-1α [96]. Supportively, HIF-1α has been suggested as a potential PKM2 metabolic upregulation factor within fibroblasts [98]. Therefore, HIF-1α can influence PKM2 and cellular reprogramming, demonstrating the intricate nature of PKM2 in cancer and beyond [98,100].

The role of PKM2 in promoting adaptation to changes in the redox microenvironment of cancer cells is also evident through its function as a modulator of the activity of the tumor suppressor protein P53. Based on the intracellular redox state, PKM2 either reduces or promotes the activity of P53. In highly oxidized environments, the tetrameric form of PKM2 suppresses P53 activity concomitant with a reduction in apoptotic cell death, while in a reduced environment, PKM2 has an opposite regulatory effect on P53 [101]. This role of PKM2 in enhancing the adaptation of cancer cells to oxidative stress increased the therapeutic interest of targeting PKM2 in conjugation with chemotherapy to mediate oxidative stress-induced cell death [75,102].

Beyond cancer, preferential PKM2 structural transformation has been observed in non-cancerous conditions. For instance, increased PKM2 dimerization has been observed in a murine model of colitis [103]. The study revealed that *Sirt5* KO mice with induced colitis exhibited increased levels of PKM2 in the dimer form. Dimeric PKM2 has also been reported to exhibit protein kinase activity [62]. A specific example of dimer PKM2 kinase activity is the activation of MEK5 transcription through the phosphorylation of STAT3 [62]. Indeed, PKM2 phosphorylates STAT3 to initiate an inflammatory response-signaling cascade and participates in increasing cancer proliferation [62,104,105]. This mechanism of interaction in which PKM2 acts as a protein kinase could further upregulate a feedforward stimulation process that promotes oncogenic cellular expansion [62]. However, these findings were later contradicted by Hosios and colleagues, who found no evidence of PKM2’s activity as a protein kinase [106]. Regardless, more work is needed to uncover the clinical significance of the role of structural configuration and kinase activity in mediating PKM2 function in tumor cells. Additionally, because the biological complexities and importance of PKM2 are not merely limited to its protein and structural interactions, a comprehensive understanding of PKM2 regulation and modification could have profound and diverse implications. Therefore, understanding how these diversified aspects of PKM2 signaling modify the outcomes of essential cellular processes such as apoptosis and inflammation could prove monumental to fully uncover its biological importance. The following will centralize around another fundamental role of PKM2, its functions, and its mechanisms of regulation.

### 3.3. PKM2 and Apoptosis

Apoptosis is the regulatory process of programmed cell death through which the homeostatic balance between cell death and proliferation is maintained [107,108]. This process is essential for normal cell turnover, immune system function, metamorphosis, hormone dependent atrophy, and chemical-induced cell death [107,108]. Alterations in apoptosis can become detrimental to the organism in a dysfunctional state [107]. This imbalance can result in biological disturbances that promote cancer, autoimmune disorders, organ damage, and many other pathological conditions [107]. The intrinsic pathway (mitochondria-associated apoptosis) and the extrinsic pathway (receptor-mediated apoptosis) are the two main pathways of apoptotic cell death. Resistance to antineoplastic agents constitutes a major obstacle in the treatment of many types of cancer.

PKM2 is a key player and regulator in the apoptotic pathways of a variety of cancers. B-cell lymphoma 2 (BCL2), a member of the BCL-2 family that is well known for its anti-apoptotic functions [91], was demonstrated to be both a direct and indirect target for PKM2. Studies in human glioblastoma multiforme (GBM) cells identified that PKM2 under oxidative stress translocates to the mitochondria, where it phosphorylates BCL-2 at threonine 69 to prevent its ubiquitination by E3 ligase and its subsequent degradation. This process is facilitated by the ATPase activity of HSP90 subunit HSP90α1, which mediates the interaction between PKM2 and BCL2. The disruption of PKM2-mediated stabilization of BCL-2 sensitized glioma cells to oxidative stress-induced apoptosis and impaired the formation of brain tumors in an orthotopic xenograft model [91]. Consistent with these findings, shRNA-mediated knockdown of PKM2 in HepG2 cells resulted in a significant reduction in BCL-2 levels, concomitant with decreased tumor growth upon subcutaneous inoculation in BALB/c nude mice [109]. Interestingly, in a recent study, the inhibition of BCL-2 using ABT737 in ovarian cancer cells resulted in lower glycolysis and PKM2 levels in a mechanism mediated by the Sirt3-HIF1α axis [110], suggesting a potential role of BCL-2 in regulating PKM2, a finding that requires further exploration.

**Figure 1:**
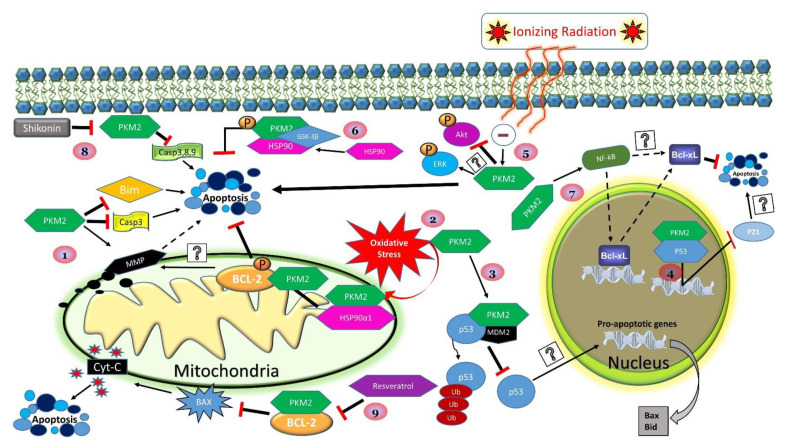
**Intrinsic role of PKM2 in apoptotic cancer cell death.** (**1**) PKM2 knockdown induces apoptosis through the stabilization of Bim, decreases in mitochondrial membrane potential (MMP), and the activation of Caspase-3 [112]. (**2**) H_2_O_2_-induced oxidative stress promotes the mitochondrial translocation of PKM2, where it is then chaperoned by HSP90α1 in order to phosphorylate and stabilize Bcl2 [91]. (**3**) PKM2 forms a complex with p53 and MDM2, a master regulator protein of pro-apoptotic genes [116]. (**4**) In the nucleus, PKM2 interacts with P53 to reduce P53 transcriptional activity and suppress P53-induced P21 transactivation [117]. (**5**) Ionizing radiation-induced apoptosis is enhanced in PKM2 knockdown cells concomitant with reduced Akt phosphorylation and increased levels of phosphorylated ERK [118]. (**6**) HSP90 mediates the complex of PKM2 and glycogen synthase kinase-3β (GSK-3β), leading to the subsequent PKM2 phosphorylation and the inhibition of apoptosis [119]. (**7**) PKM2 knockdown decreases Bcl-xL gene transcription, potentially through PKM2 stabilization of NF-κB [111]. (**8**) Modulation of PKM2 levels and/or activity alters the cleavage/activation of caspases 3, 8, and 9 to increase apoptosis [112,120]. (**9**) Inhibition of PKM2 induces apoptosis through altering the Bax/Bcl-2 ratio and the subsequent release of cytochrome c from the mitochondria [121].

PKM2’s anti-apoptotic effects seem to extend beyond its role in stabilizing BCL2 to also include other members of the BCL2 family such as BCL-XL and BIM (**Figure 1**). In a recent study, shRNA-mediated PKM2 knockdown in gastric cancer cells led to a decrease in BCL-XL expression and promoted apoptotic cell death via an NF-κB-dependent mechanism [111]. Likewise, PKM2 deficiency in hepatocellular carcinoma (HCC) cells led to an increase in apoptosis through the stabilization of BIM [112,113]. BIM is a pro-apoptotic member of the BCL-2 family of proteins and belongs to a subgroup of proteins that contains the BCL-2 homology domain 3 (BH3) only. BH3-only proteins provoke apoptosis either by direct activation of pro-apoptotic BAX/BAK or by neutralizing anti-apoptotic BCL-2 proteins including BCL-2, BCL-XL, BCL-w, MCL-1, and A-1 [114]. Regarding homeostasis, a balanced ratio of anti-/pro-apoptotic members is essential for cell survival. Alterations to this ratio by upstream apoptotic events may lead to cell death through several mechanisms including the destabilization and permeabilization of the mitochondrial outer membrane (MOMP). MOMP irrevocably commits the cell to apoptosis through a sequence of events that involves the release of several pro-apoptotic proteins from the mitochondria into the cytosol and activates a signaling cascade that leads to apoptosis [115]. Central to this critical functionality, recent studies identified BIM as a critical mediator of PKM2’s anti-apoptotic function. In HCC cells, PKM2 deficiency resulted in the stabilization of BIM, a collapse in MOMP, and the activation of the mitochondrial pathway of apoptosis [112].

Interestingly, BIM was also suggested to play a critical role in mediating the anti-apoptotic role of HSP90 in several vemurafenib-resistant melanoma cell lines [122]. These findings are also in support of the role of HSP90 in mediating the anti-apoptotic function of PKM2. Indeed, a recent study reported a direct correlation between the levels of HSP90 and PKM2 in human hepatocellular carcinoma tissue samples that were paralleled with negative clinical pathological features [119]. In vitro studies further confirmed that HSP90 enhances glycolysis, reduces apoptosis, and promotes the proliferation of HCC cells in a PKM2-dependent manner. The findings also demonstrated that, in HCC cells, HSP90 enhances the stability of PKM2 by reducing its proteasomal degradation, a process that seems to require PKM2 phosphorylation at Thr-328 [119]. Taken together, these studies identify the BCL-2 family as a major contributor to the role of PKM2 in tumor survival and resistance to therapy, but also provide new avenues for cancer treatment strategies.

In support, PKM2 inhibition using shikonin (a natural naphthoquinone extract from Lithospermum erythrorhizon, purple gromwell) increased the rate of apoptosis and induced the cleavage of caspase-3, caspase-8, and caspase-9 in human gastric cancer cells (HGC-27) [123]. In line with these findings, the effectiveness of ionizing radiation in inducing apoptosis was enhanced in PKM2 knockdown non-small cell lung cancer cells (NSCLC) [118]. The resulting increase in apoptosis was accompanied by an increase in the phosphorylation of PDK1 and GSK3β, along with AKT downregulation and increased ERK expression [118]. Another recent study revealed that resveratrol treatment of melanoma cells promoted BCL-2 degradation and increased both BAX and cytochrome c [121]. Furthermore, overexpression of PKM2 stabilized BCL-2 and prevented resveratrol’s ability to induce apoptosis [121]. These findings open new possibilities for further developments in herbal extracts and bioactive compounds targeting PKM2 as a novel approach for the disruption of cancer cell metabolism and homeostasis.

It is worth noting that several BH3-only members of the Bcl-2 family can be transcriptionally regulated by the tumor suppressor P53 [124]. As mentioned above, P53 plays a crucial role in preventing cancer through promoting apoptotic cell death. However, in more than half of human cancers, the *P53* gene is mutated into a form that exhibits oncogenic potential [125]. In malignant cells, this mutation of *P53* can promote resistance to several chemotherapeutic agents, particularly DNA-damaging drugs [125]. In a recent report, it was demonstrated that PKM2 could bind and form a complex with MDM2 and tumor suppressor P53 [116]. Dimeric PKM2 enhanced this effect, and through ubiquitination, P53 may lose its ability to transcriptionally regulate the pro-apoptotic response [116]. Through continued exploration, the relationship and interactions exerted by PKM2 on apoptotic outcomes have become more apparent. However, future studies considering the overall biological impact and metabolic consequences of PKM2 for apoptosis could reveal significant findings. Further investigating these relationships may uncover many mysteries regarding the nature of pathological disease progression and cancer immortalization.

### 3.4. PKM2 as an Inflammatory Regulator

The inflammatory response is a complex series of events where bodily injury and damaged tissues trigger the recruitment of leukocytes and neutrophils to the inflamed area [126]. Although the inflammatory response is an essential protective and healing process for the organism [126], it can also lead to autoimmune complications, with side effects such as prolonged swelling and chronic pain. Intriguingly, it has been shown that the metabolic reprogramming that occurs within the inflammatory response resembles the Warburg effect and involves PKM2 [127]. Recently, the role of PKM2 within inflammation has begun to be characterized [128,129,130,131]. In response to lipopolysaccharide (LPS) treatment, PKM2 was significantly upregulated in activated macrophages, concomitant with an increase in its dimeric form [128,130], resulting in metabolic reprogramming that promoted aerobic glycolysis. Furthermore, LPS induced the translocation of PKM2 into the nucleus and the subsequent activation of HIF-1α and the transcription of IL-1β [127,128]. Additionally, the pro-inflammatory cytokine high mobility group box-1 (HMGB1) was shown to be regulated by PKM2 through a mechanism involving metabolic reprogramming [132,133]. The findings revealed that in macrophages, PKM2 might interact with HIF1α to promote aerobic glycolysis through the activation of HIF1α-dependent enzymatic transcription, and PKM2 knockdown reduced lactate and HMGB1 release. HMGB1 is highly sensitive to redox modification, and its release can provoke cytokine induction and chemotaxis [134]. Furthermore, HMGB1 is often highly expressed in various autoimmune and inflammatory disorders. Therefore, finding safe and effective approaches for inactivating HMGB1 could counter the anti-apoptotic functions of PKM2 and may be of therapeutic value. For instance, shikonin-induced PKM2 inhibition in activated macrophages led to a reduction in the release of HMGB1 and protected against LPS-induced endotoxemia and sepsis in mice [133]. In another study, also focused on the roles of PKM2 and HMGB1 in sepsis, the authors demonstrated that macrophage inflammasome activation and aerobic glycolysis might promote the pathological progression of the disease [135]. The study also revealed that PKM2 inhibition reduces macrophage release of IL-1β, IL-18, and HMGB1, in response to a reduction in AIM2 and NLRP3 inflammasome activation.

Beyond its role in sepsis, PKM2 was also shown capable of promoting inflammation in other pathological states, a role exhibiting dependence on the structural configuration. As previously demonstrated, dimeric PKM2 has been linked to the inflammatory behavior of macrophages taken from patients with coronary artery disease (CAD) [127,130]. Dimeric PKM2 translocation into the nucleus resulted in the potential phosphorylation of STAT3 in CAD macrophages and the upregulation of IL-6 and IL-1β [130]. Xiao et al. demonstrated a potential tumor-suppressive role of eukaryotic elongation factor-2 kinase (eEF2K) in lung cancer cells through phosphorylation of PKM2 at Thr129 and the subsequent alteration in its nuclear translocation and STAT3 activation [136]. Notably, both eFK2K [137] and STAT3 [138] were demonstrated to play critical functions in the inflammatory response observed in several other inflammatory and fibrotic diseases, which may indicate a novel role for PKM2 in the pathogenesis of these diseases. As such, a broad spectrum of research has focused on delineating the role of PKM2 in inflammatory-associated diseases. Indeed, PKM2 has been shown to play a role in diabetic nephropathy [139], asthma [140], arthritis [131], osteoarthritis [141], and ischemic stroke [142]. In most cases, inhibiting PKM2 exerted beneficial outcomes, promoting its potential as a promising therapeutic target for the various mentioned conditions. Collectively, these studies identify PKM2 as a key signaling molecule in the inflammatory process in tumors and potentially in non-cancer cells [127].

### 3.5. PKM2 and Oncogenesis

PKM2’s role in aerobic glycolysis and cancer metabolism has been the focus of most of the literature and research to date [28,29,71,143]. Xenograft studies in mice injected with H1299 lung cancer cells overexpressing the mouse PKM1 or PKM2 isoforms then stably knocked down for the endogenous PKM2 revealed that PKM2 is necessary for aerobic glycolysis. Mice injected with PKM1-rescued cells showed less tumor proliferation and slower developmental time compared to mice injected with PKM2-rescued cells [71]. These results supported the theory that PKM2 plays a key role in providing tumor cells with a selective growth advantage. Follow-up studies have supported the hypothesis of a tissue-specific transcriptional switch from PKM1 to PKM2, which explains the high expression levels of PKM2 in some human cancers [144,145,146]. However, several other studies have challenged this theory [26,147], with some suggesting that the elevated level of PKM2 in tumors is rather caused by an increase in the transcription of the *PKM* gene. Notably, the study by Zhan and colleagues found that, while there is a decrease in the expression and proportion of PKM1 to total transcript variants of the *PKM* gene, a switch from PKM1 to other PK variants occurs in tumors [147]. This adds a level of complexity to the role of pyruvate kinases in oncogenesis that requires further examination as to the clinical significance and the contribution of these variants to tumor growth and survival. Regardless, over the last decade, PKM2 genetic deletion or pharmaceutical inhibition has become a central approach to the study of PKM2 in cancer.

Many studies have supported the idea that PKM2 knockdown and deletion suppress the development of cancer [71,148,149]. Conversely, a recent study found conflicting data regarding PKM2 deficiency and its ability to attenuate tumor proliferation. Indeed, PKM2 deletion in a mouse model of breast cancer accelerated tumor formation and promoted liver metastasis. Interestingly, the study also found variable PKM1 protein levels in PKM2-deficient tumors and the study concluded that PKM2 is not required by all tumor cells and that there is a differential requirement for pyruvate kinase among different tumor cell populations [148]. On the other hand, a growing body of evidence suggests that the nuclear function of PKM2 is required for the growth of some tumors, including EGFR-mutant cancers. Li and colleagues demonstrated that inhibition of the Poly (ADP-ribose) polymerase (PARP), a protein responsible for repairing damaged DNA, prevented the nuclear translocation and retention of PKM2, concomitant with a reduction in EGFR-mutant lung cancer cell growth [150]. As such, much of the current literature supports the idea that PKM2 inhibition, deletion, and suppression could prove effective in the treatment of various cancers [29,148,149]. However, it is integral to consider all angles when exploring the vast array of complexities involving cancer metabolism. The discrepancies within the results of some studies could be due to the intricate morphological variations observed in the many unique forms of cancer and experimental models utilized.

## 4. Emerging Areas of Research Involving PKM2

### 4.1. Role of PKM2 beyond Cancer

In recent times, the role of PKM2 in non-cancerous tissues has become an area of interest [25,151]. Investigations targeting compounds promoting PKM2 inhibition or activation are currently in the early developmental stages, and tissue-specific side effects of these compounds could derail the human trials [152]. Therefore, future research aimed at PKM2 tissue-specific functions could be integral to the success of PKM2 inhibitors targeting cancer or non-cancerous disorders. PKM2 is expressed in other differentiated tissues including lung, heart, pancreas, liver, and adipose tissues (both white and brown) [25,29,30,61,62,151,153]. Many studies have reported possible novel functions of PKM2 since the turn of the century [25,151,154,155]. Recently, the role of PKM2 in relation to glucose homeostasis, insulin secretion, and pancreatic function has been explored [27,155,156,157]. Therefore, it is vital to better understand the metabolic and non-metabolic functions of PKM2 within these tissues in order to develop effective therapeutic strategies with enhanced targeting efficacy.

### 4.2. PKM2 and Metabolic Homeostasis

Type 2 diabetes (T2D) has become one of the leading health epidemics worldwide. The development of T2D centralizes around β-cell dysfunction leading to decreased insulin secretion and is often accompanied by dysregulated glucose uptake in response to insulin resistance [158,159,160]. Pancreatic β islets are responsible for the release of insulin and essential to maintain glucose homeostasis. β-cell function or dysfunction is often measured by the ability of β-cells to adequately sense glucose and respond by secreting insulin in a tightly controlled process [158,161,162]. Glucose-stimulated insulin secretion (GSIS) occurs through the uptake of glucose into β-cells, which leads to an eventual rise in intracellular Ca^2+^, resulting in the subsequent insulin secretion [163]. This process is dependent upon the depolarization of the K_ATP_ channel and caused by an increased ATP/ADP ratio [163,164]. When exposed to prolonged hyperglycemic conditions, the β-cells can begin to exhibit decreased mass and impaired function, leading to impaired insulin secretion [165].

PKM2 has been demonstrated to play critical functions within the pancreas and specifically within the β-cells, in part, because of the expression pattern of both PKM1 and PKM2 within the pancreas. According to a recent study, immunohistochemistry analysis of PKM1 and PKM2 expression in the mouse pancreas revealed the significant expression of both isoforms in the islets with minimal staining in other parts of the pancreas [166]. These findings highlight a potential role in insulin secretion. Cysteine is a metabolite that has been linked to increased BMI and fat mass [90], which are biomarkers for obesity and increased risk for T2D development [167]. Plasma L-cysteine concentrations have been utilized as a potential marker of β-cell and pancreatic function [168]. Increased levels of L-cysteine reversibly inhibit glucose-induced biphasic insulin secretion and ATP production through direct binding to PKM2, leading to the dissociation of its tetrameric form and inhibition of its kinase activity. The role of PKM2 in insulin secretion was further supported in a later study where a novel signaling pathway through which PKM2 potentially promotes insulin secretion and β-cell function was identified [157]. The study demonstrated that PKM2 could promote insulin secretion and β-cell proliferation through the activation of the Wnt/CTNNB1 pathway [157].

Numerous impairments can lead to insulin resistance and dysregulated glucose uptake into skeletal muscle cells and adipocytes. Insulin resistance can occur in response to a wide variety of pathological conditions such as inflammation and oxidative stress [169]. Beyond its various roles and effects on insulin secretion, the functional association between insulin signaling and PKM2 within adipocytes is emerging as a novel area of study. Earlier studies demonstrated that exposure of 3T3-L1 adipocytes to varying levels of insulin resulted in significant increases in PKM2 mRNA levels, independent of the levels of glucose and glucosamine in the media. Moreover, pharmacological inhibition of the insulin signaling pathways using wortmannin or PD98059 to inhibit phosphatidylinositol 3-kinase (PI3K) or mitogen-activated protein kinase (MAPK), respectively, resulted in converse results, identifying insulin as an upstream modulator of PKM2 expression in adipocytes and possibly the adipose tissue [151]. Notably, our recent studies have identified PKM2 as a potential contributor to insulin resistance in the adipose tissue and made an association between alterations in PKM2 tyrosine phosphorylation at Tyr-105 and the metabolic status of rodents, primates, and humans, with increased PKM2 Y105 phosphorylation correlating with a favorable metabolic profile [154].

Beyond the reported effects on insulin signaling and responsiveness, a recent study has shown that PKM2 may play a role in brown fat adipogenesis. Isidor et al. identified that the level of PKM2 is higher in murine brown adipose in comparison to white adipose tissue [170]. Notably, PKM2 knockdown in mature brown adipocytes resulted in increased levels of thermogenic genes uncoupling protein 1 (Ucp1) and fibroblast growth factor 21 (Fgf21). The authors postulated that this may have occurred through PKM2’s ability to modify adipocyte gene expression. However, while the molecular mechanisms mediating PKM2’s function in brown fat adipogenesis are yet to be determined, these findings suggest a novel role for PKM2 in regulating body mass and energy expenditure [170], which warrants additional investigation into the contribution of PKM2 to obesity, thermogenesis, glucose homeostasis, insulin resistance, and their associated metabolic disorders.

PKM2 has also been reported for its involvement in the pathogenesis of diabetes nephropathy (DN) and its role in mitochondrial function within the renal glomeruli [25]. DN can result from mitochondrial dysfunction, leading to the increased synthesis of toxic glucose metabolites, resulting in detrimental translational outcomes [25]. Explorations targeting the contribution of PKM2 to DN and to alterations to podocyte homeostasis revealed that PKM2 activation might attenuate mitochondrial dysfunction through improved metabolic functionality and induced biogenesis [25]. Furthermore, PKM2 activation increased glucose metabolic flux and lowered toxic glucose metabolite production [25]. These findings support the idea that PKM2 activation may act as a preventative mechanism through which the progression of diabetic nephropathy could be halted [25]. However, while PKM2 activation exhibits some degree of therapeutic potential, inverse findings were discovered when PKM2 was targeted in other parts of the kidney. For instance, the reduction in PKM2 enzymatic activity in proximal tubules mediated a beneficial effect against ischemia reperfusion (IR)-induced acute renal injury (AKI). The genetic deletion of aldo-keto reductase family 1 member 1 (AKR1A1) increased the S-nitrosylation of PKM2 and reduced its enzymatic activity by inhibiting PKM2 tetramer formation. This blockade of the last step of glycolysis was suggested to shift glycolytic metabolites towards the pentose phosphate pathway and generate precursors necessary for antioxidant defense. This hypothesis was further confirmed by the deletion of PKM2 in proximal tubules. In response to IR-induced AKI, the serum creatinine and blood urea nitrogen levels were lower in the PKM2-deficient mice compared to the wild type, while the NADPH to NADP ratio was higher. The increase in NADPH levels was concomitant with a reduction in oxidized glutathione relative to its reduced form, which further confirms that the beneficial effect of deleting PKM2 against AKI is mediated by increasing the antioxidant defense capacity [171]. Given these conflicting data, further investigations into the tissue- and, perhaps, cell-specific roles of PKM2 in response to alterations to insulin secretion and glucose homeostasis may aid in our pursuit to understand the biological importance of these intricacies.

### 4.3. Regulators of PKM2

PKM2 regulation through either inhibition, activation, or deletion could offer potential as treatment options. However, in order to receive these benefits and avoid potential off-target effects, the full scope of their biological impact must be well understood. Researchers have begun focusing on how PKM2 regulation could be safely achieved [172,173]. Most current research focuses on the ability of either natural or synthetic compounds to potentially inhibit PKM2 [172,173]. While inhibitors have been thoroughly investigated, compounds acting as PKM2 activators have received far less attention. The following will focus on some of the emerging and more heavily studied compounds shown to alter PKM2 activity and functions within various experimental models of human disease.

#### 4.3.1. PKM2 Activators

**Figure 2:**
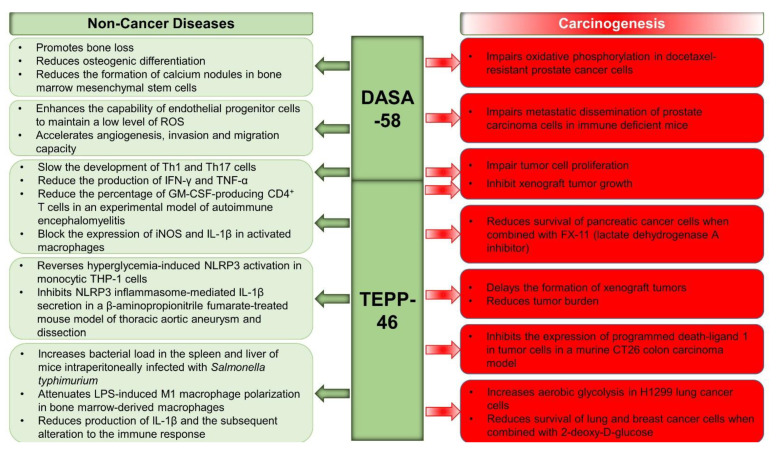
Some potential effects of PKM2 activation by DASA-58 and TEPP-46 in cancer and non-cancer diseases.

An emerging approach revolves around targeting the metabolically active tetrameric form of PKM2. In search of understanding the metabolic, kinetic, and oncogenic effects of small-molecule activators of PKM2, Anastasiou et al. investigated two separate compounds, TEPP-46 and DASA-58 (hereinafter referred to as TEPP and DASA, respectively) [174], and demonstrated that these small-molecule activators increased the level of tetrameric PKM2 enzymatic activity and increased the resistance of PKM2 to inhibition [174]. TEPP-mediated activation of PKM2 has shown promising effects in delaying the formation of xenograft tumors and reducing tumor burden [174,175] (**Figure 2**). On the other hand, DASA-mediated activation of PKM2 in vascular resident endothelial progenitor cells (VR-EPCs) promoted the activation of MAPK, AKT, and FAk signaling pathways, increased glycolysis and mitochondrial fusion, and enhanced the capability of VR-EPCs to maintain a low level of ROS. Subsequently, these effects were translated into accelerated angiogenesis, invasion, and migration capacity. Conversely, treatment with PKM2 inhibitor C3k resulted in reduced migration, invasion, and angiogenesis [176]. Likewise, the use of small-molecule activators of PKM2 resulted in reduced xenografted cancer cell proliferation [174], further confirming the detrimental effects of PKM2 activation.

In a computational high-throughput analysis of compounds with PKM2 molecular docking affinity, compound 0089-0022 was identified as a direct activator of PKM2 through kinase pocket binding [177]. Surprisingly, in vitro 0089-0022-mediated activation of PKM2 in NSCLC cells induced apoptosis in a dose-dependent manner. Moreover, 0089-0022 pro-apoptotic effects were mediated, at least in part through inhibition of AKT phosphorylation. Taken together, these studies highlight the differential behaviors of PKM2 activators and the requirement for more in-depth investigations into the proposed mechanisms of action and subsequent physiological, biochemical, and clinical outcomes of using these compounds as a potential therapeutic approach for the treatment of cancer.

Similar to cancer studies, efforts to investigate the effects of PKM2 activators under pathological conditions in non-cancerous tissues have met mixed success. As indicated earlier, DASA-mediated activation of PKM2 promoted bone loss and reduced osteogenic differentiation and the formation of calcium nodules in bone marrow mesenchymal stem cells. These effects were mediated, at least in part, through increased ROS production and alterations in mitochondrial function. Additionally, when these same cells were induced to differentiate into adipocytes, both the expression of adipogenic markers and lipid accumulation were significantly higher in DASA-treated cells [178], suggesting a pro-adipogenic role of PKM2. These findings also highlight a novel aspect of metabolic reprogramming and the critical need for developing promising strategies that target metabolism for therapy of both metabolic and non-metabolic diseases.

It is widely acknowledged that metabolic reprogramming also impacts immune cell differentiation, homeostasis, and functionality, and thus, plays a critical role in immunity and inflammation. Central to the pathophysiology of septic shock is the activation and production of inflammatory mediators. DASA- and TEPP-mediated PKM2 activation inhibited LPS-induced IL-1β and HIF-1α, as well as the expression of their downstream genes. PKM2 activation also attenuated LPS-induced M1 macrophage polarization in bone marrow-derived macrophages (BMDMs) in vitro. In vivo, PKM2 activation using TEPP resulted in increased bacterial load in the spleen and liver of mice intraperitoneally infected with S. typhimurium, possibly because of the reduced production of IL-1β and the subsequent alterations in the immune response [128].

Consistent with these findings, TEPP-mediated activation of PKM2 in a murine solid CT26 tumor model reduced the ability of macrophages, dendritic cells, T cells, and tumor cells to express programmed death-1 (PD-1) ligand 1 (PD-L1) [179] (**Figure 2**). Acting as a signaling gatekeeper, PD-L1 plays a key role in the development of immune tolerance to prevent excessive response [180], but also in blocking the development of the T cell response against tumor cells ([179]. In the same line of thought, treatment of resting CD4^+^ CD62L^+^ T cells with TEPP halted their proliferation and activation. Additionally, in vitro studies demonstrated that PKM2 activation using DASA and TEPP slowed the development of T helper 17 (Th17) cells and reduced the production of tumor necrosis factor alpha (TNF-α). Similarly, both activators decreased the development of Th1 cells and their ability to produce IFN-γ and TNF-α. In vivo, PKM2 activation using TEPP in an experimental model of autoimmune encephalomyelitis (EAE) reduced the percentage of granulocyte-macrophage colony-stimulating factor (GM-CSF)-producing CD4^+^ T cells. Together, these studies have broadened our understanding of how PKM2 may modulate autoimmunity and T cell responses [181]. However, in a recent study by Seki and colleagues looking at the effects of PKM2 activation on T cell-mediated autoimmunity in a mouse model of multiple sclerosis (MS) using both TEPP and DASA, both compounds increased the generation of GM-CSF-producing cells [182]. Given the role of GM-CSF-producing cells in EAE, the authors examined the infiltration of immune cells within different parts of the brain and found a significant increase in the accumulation of CD45^+^ cells in the periventricular regions of the brain, with fewer cells in the spinal cord, eliciting a higher encephalitogenic phenotype [182]. These differences in outcomes between studies are intriguing and warrant further investigation as the effects of PKM2 activation seem to yield different outcomes in different immune cells. In recent studies, the effects of PKM2 activation on immune responsive cells were shown to be mediated at least in part through modulation of the transcriptional regulation and activity of STAT5, HIF1α, and cMyc in CD4^+^ T cells [181], but not in natural killer (NK) cells [183] (**Figure 2**). It is worth noting that, in normal NK cells, TEPP-mediated activation of PKM2 inhibited cell growth and biosynthetic pathways and reduced the production of pro-inflammatory cytokine in response to biosynthetic pathways [181]. Together, these findings have further fueled the growing interest in targeting PKM2 as a valuable therapeutic approach to inflammatory and autoimmune diseases [181].

Given its critical role in the regulation of inflammation and the activation of immune cells, NLRP3 (NOD-, LRR-, and pyrin domain-containing protein 3) inflammasomes have attracted wide attention. For instance, NLRP3-mediated IL-1β production by macrophages exerts a key role in the development and pathogenesis of several inflammatory diseases [184], and thus, its mechanisms of regulation became an attractive therapeutic target for a spectrum of diseases. Recent studies on the role of PKM2 in mediating the activation of NLRP3 inflammasome have produced mixed results. In a study by Xie and colleagues, PKM2 deficiency and pharmacological inhibition blocked inflammasome activation as well as the cleavage and secretion of IL-1β progression of the disease [135]. These findings were further supported by Li et al. demonstrating that co-treatment of human monocytic THP-1 cells with TEPP-46 and 2-deoxy-D-glucose reversed hyperglycemia-induced NLRP3 activation [185] (**Figure 2**). Conversely, TEPP-46-mediated activation of PKM2 inhibited NLRP3 inflammasome-mediated IL-1β secretion in a β-aminopropionitrile fumarate (BAPN)-treated mouse model of thoracic aortic aneurysm and dissection (TAAD) [186]. These findings are in line with those of the O’Neill group, demonstrating that PKM2 activation in vivo using TEPP inhibits IL-1β production in an experimental model of *Salmonella typhimurium* infection [128]. Likewise, activation of PKM2 using TEPP-attenuated Sirt5-deficiency-mediated IL-1β upregulation in LPS-stimulated macrophages in vitro and in dextran sulfate sodium (DSS)-induced colitis in mice [187]. Together, these findings provide compelling evidence that PKM2 is a key regulator of the inflammatory response and prompted many scientists to explore the metabolic consequences of metabolic reprogramming using PKM2 activators in metabolic diseases. This interest is supported by recent discoveries demonstrating that PKM2 activation ameliorates kidney function in experimental models of diabetic nephropathy. Indeed, TEPP was recently found to be beneficial in reversing inflammation, alterations to renal function, as well as the associated metabolic abnormalities caused by hyperglycemia [25,139]. We anticipate the outcomes of current and future research in this area to yield novel insight into the therapeutic potential of PKM2 activator in metabolic diseases, including obesity, diabetes, and their complications.

#### 4.3.2. PKM2 Inhibitors

**Figure 3:**
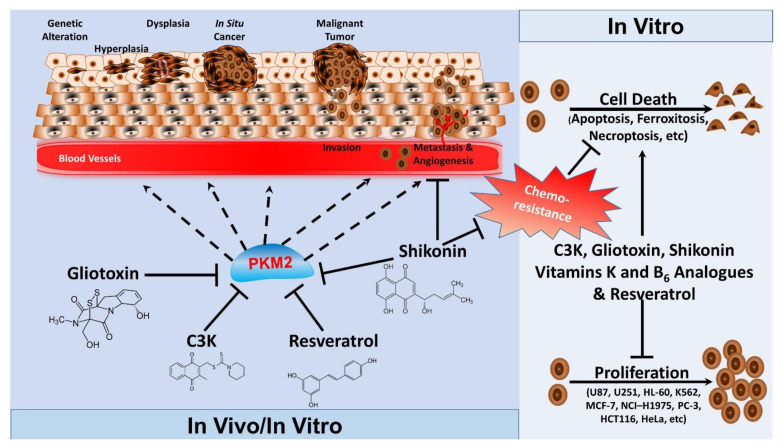
Potential in vivo and in vitro effects of selected natural and pharmacological PKM2 inhibitors.

In comparison to activators, PKM2 inhibitors have been much more heavily investigated, revealing a wide range of effects in both cancerous and non-cancerous tissue. Numerous synthetic compounds and small-molecule inhibitors such as compound C3k and analogue derivatives C3f and 3h were demonstrated to be efficient in inhibiting PKM2 [188,189] and have shown promising outcomes. Initially, Ning et al. investigated the efficacy of synthesized naphthoquinone derivatives to act as small-molecule inhibitors of PKM2 [188]. They demonstrated that compound C3k exerted a higher degree of inhibitory activity against PKM2 in comparison to the well-established natural inhibitor, shikonin. In addition, compound C3k treatment within the nanomolar range resulted in antiproliferative effects in several cancer cell lines including HeLa and HCT116 cells [189] (**Figure 3**). Beyond its role in cancer, C3k-mediated inhibition of PKM2 prevented ovariectomy (OVX)-induced bone loss and adipogenesis in vivo through modulation of the Wnt/β-catenin pathway. Furthermore, bone marrow mesenchymal stem cells treated with C3k exhibited a reduction in osteoclastogenesis, accompanied by reduced expression of several adipogenic markers including adipsin, FABP4, and PPARγ under adipogenic differentiation [178]. These findings on adipogenesis are consistent with the anti-adipogenic effects of shikonin [190,191,192,193,194] and further demonstrate that PKM2 is a key regulator of both osteogenesis and adipogenesis and suggest that targeting PKM2 might be of clinical significance in metabolic and genetic bone diseases.

Recently, Gliotoxin, a marine fungi metabolite, has been identified as a novel PKM2 inhibitor that directly binds to PKM2 to suppress its enzymatic and kinase activity. Presumably, Gliotoxin is a potentially specific PKM2 inhibitor as no changes to PKM1 enzymatic activity were observed when cell-free PK activity assay was used. Notably, preliminary studies revealed that Gliotoxin exhibits antiproliferative activity in several cancer cell lines including U87, U251, HL-60, K562, MCF-7, NCI–H1975, PC-3, HCT116, and HeLa cells [195] (**Figure 3**). However, while some effort towards uncovering the potential for synthetic compounds and their derivatives, a large number of studies have investigated various natural compounds and their analogues as PKM2 inhibitors.

#### 4.3.3. Natural Compounds

Resveratrol is a natural compound that has shown promise as a target for reduced cancer proliferation and cancer prevention [196,197,198,199]. Resveratrol has been linked to possible anticancer potential through its interaction with multiple pathways, including p53, NF-κB, SIRT1-dependent AMPK activation, and mTOR inhibition [196,200,201,202]. Recently, resveratrol has also been studied for its possible role as a PKM2 inhibitor [173]. Iqbal and Bamezai demonstrated that resveratrol could reduce PKM2 expression, inhibit mTOR, and disrupt both aerobic glycolysis and the anabolic capacity, resulting in reduced proliferation of several different cancer cell lines [173] (**Figure 3**). Conversely, PKM2 overexpression abolished these effects while shRNA silencing of PKM2 recapitulated the beneficial effects of resveratrol. This discovery could lay the foundation for how resveratrol interacts with cancer metabolism [173].

Vitamin K, a class of fat-soluble vitamers with vital physiological roles, have shown potential as anticancer agents [203,204,205,206,207,208,209,210]. Early studies have shown that menadione (also known as vitamin K_3_), a synthetic analogue of 1,4-naphthoquinone, reduced cell viability and induced a caspase-independent, but iron- and oxygen-dependent, cell death process that was termed ferroxitosis [211] (**Figure 3**). Recent studies have confirmed the cytotoxic effects of vitamin K analogues in cancer cells and demonstrated that treatment of HeLa cells with two vitamin K analogues (VK_3_ and VK_5_) resulted in reduced cell survival through modulation of glycolysis and PKM2 activity [172]. In addition to vitamin K, a recent study demonstrated that vitamin B_6_ (pyridoxine) exerts its neuroprotective effects through promoting the dimerization of PKM2, which results in Nrf2 transactivation and the upregulation of glutathione synthesis [212]. Further research efforts towards uncovering the role of vitamins K and B_6_ in regulating PKM2 activity and the translational significance of these discoveries may be integral to fully characterize their therapeutic potential as natural modulators of PKM2 activity.

Shikonin, on the other hand, and as mentioned above, is another natural compound extracted from the roots of *Lithospermum erythrorhizon* (also known as “Zicao”) that has been recognized for its potential anti-inflammatory, antimicrobial, and anticancer properties [213,214,215,216], so as for its analogue (alkannin) [172]. Treatment with either shikonin or alkannin led to PKM2 inhibition and decreased lactate production in cancer cells [172]. The results also attributed shikonin’s effects to the preferential targeting of PKM2, with no effects on PKM1 [172]. The findings identified that shikonin and its analogue could possess anticancer potential through the disruption of cancer cell glycolysis [172]. Supportively, a more recent study demonstrated that shikonin inhibits cell proliferation and survival and exacerbates cisplatin-induced cell death in T24 bladder cancer (BC) cells. These effects were postulated to be mediated, at least in part, through the direct binding of shikonin to PKM2. Notably, shikonin did not result in a reduction in overall PK activity [217]. Furthermore, shikonin treatment resulted in a reduction in actin polymerization, suggesting that shikonin could be effective in preventing cancer cell invasion and migration. However, several studies have revealed that shikonin may not be specific to only PKM2 as originally reported/postulated. Indeed, recent studies have demonstrated that shikonin is also capable of inhibiting other proteins, including protein-tyrosine phosphatase 1B (PTP1B) and the tumor suppressor phosphatase and tensin homolog (PTEN) [218]. Importantly, both PTEN [219,220] and PTP1B [221] have been identified in various roles essential for tumorigenesis. More recently, the effects of shikonin on hepatocellular carcinoma (HCC) were explored. Shikonin treatment inhibited PKM2, resulting in apoptosis induction, inhibited glycolysis, and reduced proliferation [222]. In another recent pancreatic ductal adenocarcinoma (PDAC) study, the biomechanical role of PKM2 in the regulation of Ca^2+^-dependent cell death was explored. The authors demonstrated that through depleting ATP, reducing glycolysis, increasing the level of free Ca^2+^, and inhibiting the capacity of the plasma membrane calcium pumps (PMCA), shikonin inhibition of PKM2 resulted in increased cell death and reduced metastatic potential and proliferation in human Mia PaCa-2 cells and PDAC cells [223]. While diversified and promising, the effects of shikonin are not limited only to carcinogenesis.

**Figure 4:**
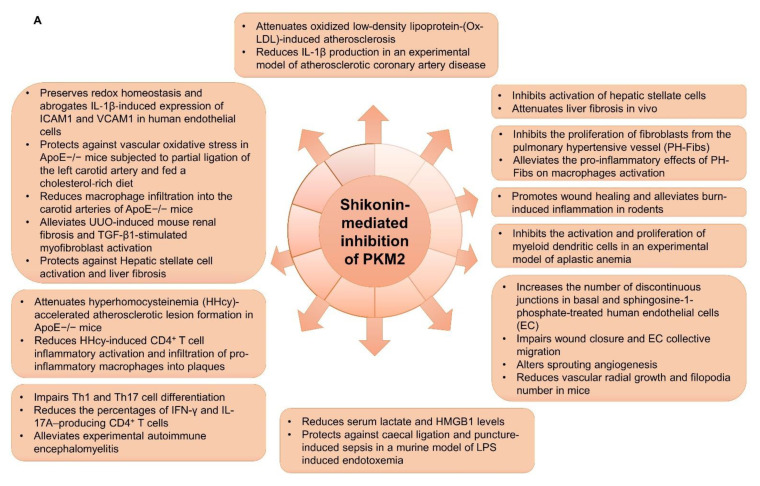

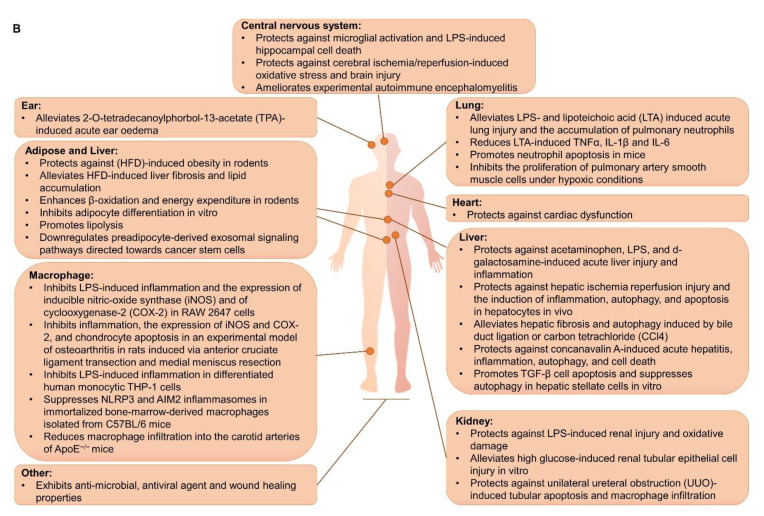
Effects of shikonin-mediated inhibition of PKM2 (**A**) and some other reported effects (**B**) not necessarily linked to PKM2.

Beyond cancer, a wide variety of potentially beneficial and therapeutic effects of shikonin have been revealed. As reviewed by Chuanjie et al. numerous studies have identified that shikonin may possess anti-inflammatory capabilities through regulatory actions exerted on a wide variety of proposed signaling molecules and pathways involving NF-κB, TLR4, ERK, JNK, MAPKs, STAT3, cytokines, and more [224]. While limited, the involvement of PKM2 in the anti-inflammatory actions of shikonin has been investigated. Wang et al. demonstrated that treatment with shikonin resulted in a reduction in serum lactate and HMGB1 levels and protected against cecal ligation and puncture (CLP)-induced sepsis and in a murine model of LPS-induced endotoxemia [133] (**Figure 4A**). Additionally, shikonin-mediated inhibition of PKM2 abrogated oxidized low-density lipoprotein-(Ox-LDL)-induced atherosclerosis and the expression of HIF-1α target genes. Furthermore, shikonin treatment led to a reduction in IL-1β production in an experimental model of atherosclerotic coronary artery disease [225]. These anti-inflammatory effects of shikonin-mediated inhibition of PKM2 were further confirmed in several experimental models of human inflammatory diseases [226,227,228,229] (**Figure 4A**). For instance, shikonin treatment promoted wound healing and alleviated burn-induced inflammation in rodents [229], preserved redox homeostasis and abrogated IL-1β-induced expression of ICAM1 and VCAM1 in human endothelial cells, and protected against vascular oxidative stress in apolipoprotein E-deficient (ApoE^−/−^) mice subjected to partial ligation of the left carotid artery and fed a cholesterol-rich diet. Additionally, shikonin reduced macrophage infiltration into the carotid arteries of ApoE^−/−^ mice [230], alleviated UUO-induced mouse renal fibrosis and TGF-β1-stimulated myofibroblast activation [231], and inhibited the proliferation of fibroblasts from the pulmonary hypertensive vessel (PH-Fibs) and the subsequent activation of macrophages [226]. Furthermore, mice treated with shikonin were protected against hepatic stellate cell activation and liver fibrosis [228]. Shikonin was also shown to suppress the inflammatory immune response through modulation of glycolysis and PKM2 activity. In an experimental study of severe aplastic anemia, shikonin suppressed the activation and proliferation of myeloid dendritic cells [232]. Likewise, PKM2 inhibition using shikonin impaired Th1 and Th17 cell differentiation, reduced the percentages of IFN-γ and IL-17A-producing CD4^+^ T cells, and ameliorated experimental autoimmune encephalomyelitis [233,234]. Shikonin treatment was also shown to be effective in reducing hyperhomocysteinemia (HHcy)-induced CD4^+^ T cell activation and infiltration of pro-inflammatory macrophages into plaques [235] and in attenuating HHcy-accelerated atherosclerotic lesion formation in ApoE^−/−^ mice [236] (**Figure 4A**). Future efforts towards uncovering the anti-inflammatory potential of shikonin may prove significant. Further promoting the importance of future investigations, shikonin has shown significant promise as an antimicrobial and antiviral agent, as well as a wound healing promoter [224,229,237,238,239,240,241,242] (**Figure 4B**). Nevertheless, it remains to be determined whether these beneficial properties of shikonin are mediated through modulation of PKM2 activity. Similarly, it remains unclear whether the anti-obesogenic and antidiabetic effects of shikonin are mediated through inhibition of PKM2; however, the health-promoting effects of shikonin may uncover novel roles of PKM2 in the pathogenesis of various metabolic and non-metabolic human diseases. Recent studies have shown that shikonin exerts protective effects against high fat diet-(HFD) induced obesity in mice [243,244] and rats [245]. Shikonin also alleviated HFD-induced hepatic lipid accumulation, enhanced β-oxidation and energy expenditure in mice [243,246], and prevented HFD-induced liver fibrosis in rats [247]. Additionally, in vitro studies have repeatedly demonstrated that shikonin and its derivatives inhibit adipocyte differentiation [190,191,192,193,194,248,249], promote lipolysis [245], and downregulate preadipocyte-derived exosomal signaling pathways directed towards cancer stem cells [248] (**Figure 4B**).

Consistent with the anti-inflammatory outcomes of shikonin-mediated inhibition of PKM2, in vitro and in vivo studies have recognized shikonin for its protective roles against oxidative stress and its associated tissue and organ damage in a number of experimental models of human diseases. For example, shikonin was shown to be capable of alleviating LPS-induced renal injury and oxidative damage [250], high glucose-induced renal tubular epithelial cell injury in vitro [251], and unilateral ureteral obstruction (UUO)-induced tubular apoptosis and macrophage infiltration [252]. Similar findings were obtained in experimental models of cardiac dysfunction [253], acute lung [254,255,256], liver [257,258,259,260,261,262], and ear injuries [263]. Shikonin also protected against cerebral ischemia/reperfusion- and autoimmune encephalomyelitis-induced brain injury through reducing oxidative stress [264,265] and protected microglial cells against LPS-induced cell death [266] (**Figure 4B**).

#### 4.3.4. PKM2 microRNAs

##### microRNA Introduction

In recent times, gene expression regulation by a specific class of small, non-coding RNA molecules known as microRNAs (miRNAs) has drawn great interest due to the diverse biological effects and modifications of miRNAs. The regulatory capability and transcription of miRNAs is propagated through a complex series of events [267,268]. The dominant canonical pathway begins in the nucleus, where primary-miRNA (pri-miRNA) transcription occurs by RNA polymerase II (Pol II) generating the initial hairpin structure of the gene [267]. This is followed by micro-processing by Drosha (RNase III protein) and DiGeorge syndrome critical region 8 (DGCR8) into pre-miRNA for export into the cytoplasm through a nuclear pore transport complex involving exportin 5 (EXP5). Once in the cytoplasm, RNase III protein and endonuclease, Dicer, binds to the 5′ end and cleaves the pre-miRNA into a small 22-nucleotide RNA duplex containing a guide and passenger strand. Sequentially, highly regulated argonaute (AGO) proteins are loaded with the RNA duplex to form the RNA-induced silencing complex (RISC). The RISC fully matures upon removal of the passenger strand of mRNA, allowing it to bind to and degrade mRNA [267]. Ultimately, this degradation can result in reduced protein translation. Dysregulation of this process can lead to profound metabolic and pathological consequences [268]. Numerous alterations to miRNAs, such as single-nucleotide polymorphisms (SNPs), methylation, and stabilization, could, in some cases, be pathologically advantageous for the development of cancer [268]. Importantly, miRNAs could prove invaluable as prognostic and diagnostic biomarkers in a wide variety of cancers, potentially improving our understanding of clinical adversities to treatment and preventative care [269,270,271]. Recent explorations continue to pioneer our understanding of the diverse roles of miRNAs and gene silencing in cancer research. In this review, we sought to comprehensively analyze the collective findings of research targeting the specific interactions and effects of non-coding RNAs including miRNA, lncRNAs, and circRNAs on PKM2 functions in cancer and beyond.

##### miRNAs in Cancer

In search of identifying novel tumor suppressors against B-cell chronic lymphocytic leukemia cells, two distinct miRNA genes, *miR-15a* and *miR-16-1*, were discovered [272]. Further investigations revealed that these genes were frequently dysregulated in clinical cases of chronic lymphocytic leukemia and that they could repress BCL-2 and induce apoptosis [273,274]. Following these discoveries, the role of miRNA dysregulation in cancer has become increasingly more evident. As displayed in the ever-expanding scope of miRNA cancer research, alterations in miRNA expression can generate either tumor suppressive or oncogenic effects. Variations in miRNA expression can result in consequential effects on the development, progression, and metastatic potential of cancer [275]. Furthermore, a wide array of cancer research has investigated the effects of various miRNAs on PKM2 expression and activity, as well as their ability to modulate tumorigenesis. Through targeting either tumor suppressors such as P53 [276] or oncogenes such as c-Myc [9], miRNAs can have profound effects on the proliferative and metabolic outcomes of cancer. For instance, miR-33b [277], miR-let-7a [278], and miR-143 and 145 [279] can act to downregulate c-Myc and lead to antitumorigenic effects [9]. Critical revelations regarding the intricacies of tumorigenesis could occur through further investigating the emerging roles of miRNAs in cancer development and proliferation.

##### miRNAs Regulation of PKM2 in Cancer

The research on miRNAs continues, with a large body of research revealing the dynamic interactions between miRNAs and PKM2 in tumorigenic processes. Collectively, a large majority of the investigated miRNAs suppress PKM2 activity, resulting in antitumorigenic effects in a wide variety of cancers [280,281,282,283,284,285,286]. Through direct binding to the 3′UTR region of the *PKM* gene, miRNAs can effectively modulate several metabolic and biological processes. Although PKM1 and PKM2 share the same 3′UTR region [287], most research on miRNA has focused on PKM2 due to its significant contribution to cancer pathology. As outlined in the following section, PKM2 regulation by miRNAs results in alterations to biomarkers evident of changes in glycolysis [280,281,282,284,288,289], metastasis [282,290,291], and cellular proliferation [286,292,293]. However, because the expression of both PKM2 and miRNAs can be restricted to specific tissues [294] and/or specific conditions, understanding the tissue and function specificity of miRNAs could prove invaluable to the implementation and translation of miRNA research into therapeutic application. Here, we provide an overall review of miRNAs to regulate PKM2 and modify biological outcomes in both an oncogenic and tumor-suppressive fashion.

*miRNAs/PKM2-Mediated Metabolic Reprogramming in Cancer*. As previously described, PKM2 regulation and modification can result in alterations in glucose utilization, lactate production, relative ATP levels, and many other aspects of glycolysis in cancer cells. Nevertheless, only miR-214 has been reported to potentially regulate PKM2 and glycolysis in a manner promoting oncogenesis. In a study by Zhang et al. the downregulation of miR-214 in NSCLC cells resulted in reduced PKM2 expression, decreased glucose consumption and lactate production along with halted cell proliferation [295]. Interestingly, the most extensively investigated cancer research model of miRNA/PKM2 interaction has centralized around the effects of alterations in the expression of various miRNAs on HCC. Several miRNAs have been shown to target PKM2 in HCC. For example, miR-122 can effectively reduce PKM2 expression at both the protein and mRNA level [287], decrease glycolysis [287,288], reduce proliferation [296], and increase apoptosis [288,296]. In addition, clinical analysis of HCC tissue samples revealed that reduced expression of miR-122 was correlated with poor three-year survival outcomes [296]. These findings suggest that miR-122 overexpression could inhibit growth and the metastatic potential of HCC. Likewise, miR-122 has been shown to reduce PKM2 expression, glucose uptake, and lactate production in human colon cancer cells including HCT-116 and HT-29 [280]. Numerous other miRNAs, such as miR-374b [297], miR-199a [281], miR-338-3p [282], and miR-491-5p [298], have demonstrated similar metabolic reprogramming effects in HCC models. These effects seem to be generalizable to other types of cancers as similar outcomes were also observed in gallbladder carcinoma [283], clear-cell renal cell carcinoma (ccRCC) [284], breast cancer (BC) [299], ovarian cancer [286,300,301], glioma [302], NSCLC [303], and melanoma [293]. In addition to their role in metabolic reprogramming, many miRNAs can also regulate other aspects of carcinogenesis such as cell migration [282,283], proliferation [112,284,286,289,293,296,298], tumor formation [112,301], cell death [288,291,292,296,304,305], and resistance to chemotherapy [280,297].

*miRNAs’ Effects on Apoptosis and Proliferation.* Emerging strategies show that a wide variety of both conventional and unconventional therapies can be effective at inducing apoptosis and ultimately cell death in cancer [306]. As indicated above, aberrant regulation of miRNAs can alter the apoptotic process, paralleled with a reduction in PKM2 expression in several cancer models. This tumor-suppressive pattern of the apoptotic machinery has been demonstrated in numerous experimental models of human cancer including miR-122 in HCC [288,296], miR-326 and miR-let-7a in cervical cancer [291,305], and miR133a and finally miR-133b in tongue squamous cell carcinoma (SCC) [292]. On the other hand, miR-4417 acted as an oncogene, where it decreased the expression of TRIM35, a protein previously identified as a tumor suppressor [307], promoted PKM2 Tyr-105 phosphorylation, increased proliferation, and reduced apoptosis in an HCC model [308]. In many cases, the effects on apoptosis were in direct accordance with reductions in cell proliferation [291,292,296,305] (**Table 1**).

Beyond their role in apoptosis, several PKM2 regulatory miRNAs were shown to modulate other signaling pathways such as autophagy. Autophagy is a vital process that often functions to catabolize cellular components in an attempt to reestablish homeostasis [309]. However, autophagy can become dysregulated in cancer and can either promote or inhibit tumorigenesis [310]. In an in vitro pancreatic ductal adenocarcinoma (PDAC) model, miR-124-induced downregulation of PKM2 expression was accompanied by a reduction in autophagic flux and enhanced gemcitabine-induced apoptosis [304]. However, an increase in the PKM1 to PKM2 ratio through miR-124’s suppression of PTBP1 resulted in increased oxidative stress, apoptosis, and autophagy in an in vitro CRC model. Similarly, miR-133b and miR-1 decreased PKM2 levels through a mechanism of indirect regulation mediated by decreased PTBP1 levels, resulting in autophagy induction, increased ROS and ATP, and reduced xenograft tumor volume in a CRC model [311]. More research targeting the differential roles of miRNAs in modulating PKM2 and autophagy could reveal vital aspects of carcinogenesis regarding the promotion or disruption of cancer cell homeostasis.

Many miRNAs that downregulate PKM2 are also capable of reducing tumor growth and proliferation. This includes miR-122 [312] and miR-139-5p (gallbladder cancer) [283], miR-199a (HCC) [112], miR-let-7a in (GC and cervical cancer) [291,313], miR-124 [289,314], miR-133b, miR-1 [311], miR-137 and miR-340 (CRC) [289], miR-184 (ccRCC) [284], miR-152 (breast cancer) [285], miR-338-3p (ovarian cancer) [286], miR-625-5p (melanoma) [293], and miR-491-5p (osteosarcoma and HCC) [298,315]. Inversely, in NSCLC, miR-214 exhibited an oncogenic role as it increased PKM2 mRNA and protein levels and promoted glucose utilization, lactate production, and cellular proliferation [295]. Beyond their effects on proliferation and apoptotic cell death, miRNAs have also been shown to exert varied effects on aspects important to tumor expansion, migration, and metastasis, as outlined in **Table 1** [282,283,290,291,298].

*miRNAs Effects on Metastatic Potential.* Cancerous lesions spread through their ability to proliferate and invade the surrounding tissue through a process known as metastasis. This process can allow tumors to spread throughout various interconnected tissues in the body. While killing or removing cancer completely is often the primary treatment goal, inhibiting its capacity to spread remains a central concern of healthcare providers. Studies on the role of PKM2-regulating miRNAs reveal their potential in disrupting the metastatic tumor initiation and progression of numerous forms of cancer. As reported by Lu et al. miR-122 can disrupt gallbladder cancer cellular malignancy through preventing TGF-β-induced epithelium mesenchymal transformation and downregulation of PKM2 expression [312]. Similarly, overexpression of miR-139-5p resulted in reduced gallbladder cancer cellular proliferation, migration, and invasion. These effects were also mediated through suppression of PKM2 expression [283]. In another study, a gene-profiling analysis in human HCC revealed a strong association between higher expression levels of circMAT2B and glycolysis. Remarkably, overexpression of circMAT2B increased glycolysis both in vitro and in vivo and promoted tumor growth and metastasis in vivo through modulation of miR-338-3p activity and its downstream target PKM2 [282]. On the other hand, recent studies have found a strong correlation between miR-let-7a and PKM2 expression and clinical characteristics of cervical cancer [291]. In this study, miR-let-7a expression levels in tissue samples of cervical cancer negatively correlate with PKM2 expression and clinicopathological indicators of the disease [291]. Conversely, the overexpression of miR-let-7a suppressed the cell migration and invasion of two established in vitro models of human cancer of the cervix uteri (SiHa and HeLa cells) [291]. Similar findings were observed using gastric cancer cells (SGC-7901 and BGC-823), where increased levels of miR-let-7a led to a reduction in PKM2 levels through modulation of the transcriptional activity of c-Myc and hnRNPA1 [313]. Lastly, miR-148a and miR-326 were both shown to reduce colonic expansion and the overall metastasis of thyroid cancer by downregulating PKM2 levels [290]. Taken together, these findings clearly demonstrate that miRNAs can play multifactorial roles through their effects on tumor metabolism, proliferation, apoptosis, and autophagy.

*Overcoming Chemotherapy Resistance with PKM2-Associated miRNAs.* While progress towards safer and more effective chemotherapeutic treatments has progressed, many hurdles still hinder the success and desirability of these therapies [324]. Issues facing chemotherapeutics often centralize around collateral cytotoxicity and successful target delivery [324]. Cancer researchers continue to explore innovative solutions to address these problems. For instance, PKM2 inhibition has been demonstrated to enhance the response of bladder cancer cells to cisplatin [217] and THP [325]. Consequently, several PKM2-regulating miRNAs were demonstrated to be capable of enhancing the susceptibility of cancer cells to chemotherapy (**Table 1**). For example, the overexpression of miR-122 inhibited PKM2 expression and resulted in the sensitization of human resistant colon cancer cells to 5-Flourouracil treatment [280]. These chemo-sensitizing effects were recapitulated in a xenografted murine model, showing a significant reduction in tumor volume in response to miR-122 overexpression upon treatment with 5-Flourouracil. In a similar HCC model, Zhang et al. identified that through its effects on the miR-374b/hnRNPA1/PKM2 axis, miR-374b overexpression antagonized glycolysis, resulting in sorafenib treatment re-sensitization. In addition, clinical observations of sorafenib-resistant HCC patients identified an inverse association between miR-374b and the upregulation of hnRNPA1 and PKM2 [297]. Likewise, the overexpression of miR-124 in a pancreatic cancer model enhanced the gemcitabine induction of apoptosis, effects possibly attributable to the miR-124/PTBP1/PKM2 axis. In a breast cancer model, enhanced expression of miR-152 may sensitize cancer cells to paclitaxel therapy through modulation of the β-catenin pathway [285]. Finally, miR-133b may overcome radio-resistance through reduced glucose uptake and lactate production [303]. Therefore, further research aimed at uncovering the clinical relevance of miRNAs in cancer exhibits profound multifaceted potential, as evidenced by the diversified aspects of carcinogenesis affected by their expression.

##### Regulation of PKM2 by miRNAs in Non-Cancerous Tissues

Aside from its roles in cancer metabolism and pathology, PKM2 is also expressed in various tissues, suggesting a possible role for PKM2 beyond cancer. As indicated in the previous sections, several reports point to the possible contribution of PKM2 to metabolic and inflammatory disorders [127] and identify PKM2 as a potential therapeutic target. Therefore, in the following section, we will discuss the regulation of PKM2 by microRNAs and the associated effects under healthy and disease conditions.

*Liver.* To our knowledge, PKM2 has not been reported to be expressed in adult healthy livers, but it can be expressed under abnormal liver disease-associated conditions such as steatohepatitis, non-alcoholic fatty liver [326], cirrhosis, and liver cancer [327]. Remarkably, the expression of both PKM2 and miR-122 seems to be responsive to diet. Notably, in an experimental model of high-fat diet-induced obesity in mice, PKM2 expression was induced in hepatocytes [328], suggesting that PKM2 may play a role in the pathogenesis of obesity-associated liver dysfunction. Overfeeding of geese resulted in decreased miR-122 levels within the liver [329]. This point is relevant because it introduces the hypothetical concept of targeting PKM2 as a potential strategy to prevent fatty liver diseases. In support of this hypothesis, miR-122 has been identified as one of the most abundantly expressed microRNAs within healthy livers [330] and was demonstrated to inversely correlate with the expression of PKM2 under pathological conditions [287,296]. This hypothesis is further supported by the findings of the Zhiliang Gu group demonstrating that miR-122 depletion in chicken hepatocytes increased PKM2 mRNA levels [331]. The clinical significance of these findings is yet to be determined, although recent observations of liver tissues obtained from obese females with non-alcoholic fatty liver disease were shown to exhibit decreased levels of miR-122 [332], which were concomitant with the increase in PKM2 expression [326] (**Table 2**). Collectively, these studies point to the possible role of liver miR-122 in the regulation of lipid and glucose metabolism and suggest that the dysregulation of miR-122 expression could contribute to the pathophysiology of fatty liver disease. Therefore, identifying compounds targeting the miR-122/PKM2 axis might be of therapeutic value.

*Central Nervous System.* Unlike the liver, both the brain [49] and spinal cord [333] express high levels of PKM2. However, the function of PKM2 within the central nervous system is largely unexplored. A recent study highlighted the possible beneficial effects of targeting PKM2 against brain damage in a mouse model of ischemic stroke. Administrating recombinant PKM2 (rPKM2) shortly after stroke resulted in an acute neuroprotective effect, while late administration of rPKM2 was positively associated with improved functional recovery [334]. In the same model, a decrease in PKM2 expression was associated with increased miR-19a levels paralleled with decreased glucose uptake. Inhibition of miR-19a in oxygen-glucose-deprived (OGD) neuronal cells, a model used to mimic hypoxic neuronal damage, restored PKM2 expression and reduced apoptotic cell death. These studies suggest that increased miR-19a could exacerbate cerebral ischemic injury, while inhibiting miR-19a might be of therapeutic value in ischemic stroke therapy [335]. In addition, recent studies have shown increased levels of miR-143 in rats with ischemic brain injury and in cultured neurons deprived of oxygen and glucose (OGD). This increase was accompanied by a decrease in the mRNA and protein levels of HK and PKM2 as well as a reduction in glucose uptake. Inhibiting miR-143 protected cultured neurons against OGD-induced caspase 3 activity and cell death, at least in part, through increased glucose uptake [336] (**Table 2**). However, further research is needed to better elucidate whether regulation of PKM2 mediates the anti-apoptotic effects of miR-143. Additionally, research investigating the potential of targeting PKM2 using miRNAs as a novel therapeutic approach to treat cerebral ischemic injury would provide valuable insights.

*Cardiovascular Diseases*. In addition to its role in maintaining CNS homeostasis, miR-143 has been also reported to be associated with endothelial cell dysfunction and plaque formation. The expression of miR-143 was shown to be upregulated in human atherosclerotic plaque specimens compared to normal arteries, concomitant with a decrease in mRNA levels of glycolytic enzymes including PKM2 and HK. These findings suggest a link between glycolysis and atherosclerotic plaque development. Overexpression of miR-143 in endothelial cells decreased ATP and lactate production, as well as glucose uptake. Although the effects of miR-143 on PKM2 are yet to be determined, Xu and colleagues demonstrated that miR-143 directly binds to HK, leading to its degradation [337].

Recently, Paola Caruso et al. demonstrated that an increase in glycolysis promotes pulmonary artery endothelial cell growth, a hallmark of pulmonary arterial hypertension (PAH) [338]. This increase in glycolysis was accompanied by a reduction in miR-124 levels in blood outgrowth endothelial cells (BOECs) obtained from patients with PAH [338]. However, bone morphogenetic protein receptor 2 (BMPR2), a protein that is normally mutated in heritable PHA and considered to be the major cause of PHA by promoting the overgrowth of endothelial cells [338] and artery smooth muscle cells [339], was suggested to regulate miR-124 expression. Silencing BMPR2 reduced miR-124 expression, revealing a new possible mechanism for BMPR2 in mediating PHA. Overexpression of miR-124 reduced PTBP1 and glycolytic enzymes including PKM2 to normal levels. These effects were associated with a reduction in cell proliferation and enhanced mitochondrial activity. Likewise, silencing PTBP1 resulted in a phenotype similar to that observed in overexpressing miR-124, suggesting that miR-124 action on glycolysis and PKM2 is mediated through inhibition of PTBP1 [338] (**Table 2**). In conclusion, targeting the BMPR2/miR-124/PKM2 axis may restore glycolysis and normalize proliferation rates in cells critical to pulmonary vasculature remodeling, which could serve as a novel therapeutic option for PHA treatment.

#### 4.3.5. Long Non-Coding RNA Targeting of PKM2

Similar to miRNAs, long non-coding RNA (lncRNAs) were once thought to be a part of the “junk DNA” with no known functions [343,344]. Nonetheless, while comprising only an infinitesimal portion of encoded genomic DNA, a growing body of literature has focused on the diverse impact and roles of lncRNAs in both healthy and diseased states [344]. LncRNAs differ from miRNAs in several ways, including being composed of 200 or more nucleotides, making them considerably larger [344]. LncRNAs lack open reading frames and often rely on their flexibility and conformational structure in order to exert their functions. Important to cancer research, lncRNAs can function to regulate aspects of metastasis, gene expression, chromatin remodeling, DNA methylation, and differentiation [344]. lncRNAs were demonstrated to interfere with the mRNA regulatory capability of miRNAs through “sponging” [344,345]. Furthermore, lncRNAs’s ability to sponge miRNAs can lead to changes in PKM2 expression levels and oncogenic outcomes [304,346]. Beyond this action, numerous other effects including both tumor-suppressive [66,89,347,348] and oncogenic functions [349,350,351,352,353,354] have been observed (**Table 3**). Therefore, the following will shed light on the emergence of lncRNAs in cancer research and the impact that they can have on tumor metabolism through regulating PKM2.

##### Tumor-Suppressive PKM2/lncRNAs

Tumor-suppressive roles of lncRNAs were only observed in liver and prostate cancer experimental models. Several studies focused on lncRNA MEG3 and how it can possibly inhibit cell proliferation [89] and reduce the migration and metastasis potential of hepatocellular carcinoma cells [66]. Regarding migration, the expression of lncRNA MEG3 was promoted following arsenic trioxide treatment and was negatively correlated with PKM2 mRNA and protein levels [66]. Consequently, HCC migration and epithelial to mesenchymal transition (EMT) capabilities were reduced. In an experimental model of liver cancer, MEG3 was shown to promote miR-122 expression, leading to a reduction in PKM2 expression and altered nuclear translocation capabilities [89]. This further resulted in PTEN-dependent β-catenin inhibition and degradation, as evidenced by decreased binding of β-catenin to its downstream target LEF/TCF4. The effect of MEG3 on β-catenin activity was further validated using luciferase activity assay of LEF/TCF4, where the overexpression of MEG3 reduced LEF/TCF4 activity. The reduced β-catenin activity was suggested to be mediated through several mechanisms including the modulation of PKM2 function and subcellular distribution. Overexpression of MEG3 reduced the interaction between phosphorylated PKM2 and β-catenin and the subsequent nuclear localization of β-catenin. Additionally, MEG3 promoted the ubiquitination and degradation of β-catenin via a PTEN-dependent mechanism. These effects resulted in reduced cellular proliferation and tumor growth both in vitro and in vivo [89]. Similarly, ectopic expression of long intergenic non-coding RNA (lincRNA) 01,554 in HCC led to a reduction in cell growth and colony formation, through the reduction of PKM2 levels by ubiquitin-mediated degradation, and inhibition of Akt/mTOR signaling pathway to reduced aerobic glycolysis [347]. Modulation of the Akt/mTOR signaling pathway appears to mediate the suppressive effects of other lncRNAs on PKM2 expression. For example, lincRNA-p21 indirectly downregulated PKM2 expression in prostate cancer, through the PTEN/AKT/mTOR pathway [348]. Additionally, downregulation of lincRNA-p21 increased glucose consumption, lactate production, pyruvate levels, proliferation, and tumorigenic potential. However, while some examples of tumor-suppressive PKM2/lncRNAs have been identified, the vast majority act to promote oncogenic outcomes (**Table 3**).

##### Oncogenic PKM2/lncRNAs

As demonstrated in a CRC study, lncRNA-FEZF1-AS1 increased PKM2 stability and protein levels through direct binding, and it increased lactate production and PK activity [349]. As previously mentioned, many PKM2/lncRNAs may act to promote some aspect of carcinogenesis in a variety of cancers. In a CRC model, lncRNA MAF-AS1 may sponge miR-147b to increase PKM2 mRNA levels, proliferation, cell cycle progression, and cell invasion [346]. In liver cancer, lncRNA HULC increased PKM2 protein levels. HULC also increased LC3II and Sirt1, resulting in elevated levels of autophagy and increased cell growth [7]. Silencing of LncRNA XLOC_006390 resulted in decreased PKM2 mRNA and protein levels and promoted tumorigenic potential in an experimental model of cervical cancer. In addition, XLOC_006390 may act as a negative regulator of miR-331-3p and miR-338-3p. Several other PKM2/lncRNAs including lncRNA RPPH1 in BC [10] and lncRNA H19 ovarian cancer [351] were also shown to promote carcinogenesis, suggesting their oncogenic function. Interestingly, red ginseng extract ginsenoside 20(S)-Rg3 has been shown to inhibit the Warburg effect and halt cancer cell growth through the lncRNA H19/miR-324-5p/PKM2 axis [351], where treatment with 20(S)-Rg3 indirectly enhanced the suppression of PKM2 by miR-324-5p. Beyond lncRNAs, linc-ROR (PDAC) [304] and linc-00689 (glioma) [350] both exhibit oncogenic and PKM2 regulatory potential. Silencing of ROR decreased PKM2 protein levels and enhanced gemcitabine-induced apoptosis [304]. On the other hand, overexpression of ROR increased autophagy and, together, the effects show that ROR may sponge miR-124 (a negative regulator of PTBP1) to increase PKM2 levels and confer chemoresistance [304]. In a glioma model, silencing of linc00689 suppressed glycolysis, cell proliferation, migration, and invasion [350]. Moreover, the linc00689/miR-338-3p*/PKM2* axis may have oncogenic potential and modulating this axis could prove effective in glioma therapy [350]. Taken together, these studies reveal that through silencing or overexpressing lncRNAs, important aspects of carcinogenesis such as proliferation, glycolysis, apoptosis, autophagy, and metastatic potential may be modified towards beneficial outcomes (**Table 3**). Thus, lncRNAs should be carefully considered for the future development of novel approaches to cancer therapeutics and application.

##### PKM2/lncRNAs in Non-Cancer Diseases

While the role of some PKM2/lncRNAs in cancer has been extensively studied, the physiological function and molecular mechanisms by which PKM2-regulated lncRNAs act in non-cancer diseases remain to be elucidated. Recently, using an experimental model of Alzheimer’s disease (AD), lncRNA RPPH1 was demonstrated to exhibit a protective effect against ER stress and apoptosis induced by extracellular amyloid (Aβ) deposits in SH-SY5Y cells. These effects were mediated, at least in part, through downregulating miR-326, which acts as a suppressor of PKM2 expression. The overexpression of miR-326 was shown to directly inhibit PKM2 expression and upregulate ER stress and apoptotic markers including GRP78, CHOP, and caspase-12, whereas lncRNA RPPH1 overexpression decreases ER stress and promotes survival by sponging and counteracting the inhibitory role of miR-326 on PKM2 [357] (**Table 4**). Of note, miR-326 was previously shown to modulate ER stress and mitochondrial fission [305], both of which are well-established contributors to the pathogenesis of neurodegenerative diseases including Alzheimer’s disease [364]. Together, these findings suggest that lncRNA RPPH1 could serve as a valuable therapeutic approach in neurodegenerative disorders upstream of PKM2 [357]. Additionally, given PKM2’s role in metabolic regulation, further characterization of these PKM2-associated lncRNAs will provide a better understanding of lncRNA-mediated gene regulation in the pathogenesis of a variety of metabolic and non-metabolic diseases. In a recent study, upregulation of lncRNA-Malat1 was suggested to exert several beneficial effects on pancreatic β-cell homeostasis and to prevent lipotoxicity-induced β-cell dysfunction through modulating the Ptbp1/PKM2 axis [365] (**Table 4**). However, there is still more research needed to explore the role of PKM2/lncRNAs in health and disease.

#### 4.3.6. CircularRNA

The metabolic and overall cellular consequences that can occur in response to miRNAs regulation of PKM2 are becoming increasingly apparent. However, the complete perspective regarding the intricate nature of intracellular factors by which miRNAs exert control over cellular transformation remains elusive. Beyond the metabolic and oncogenic contributions of miRNAs and lncRNAs, other non-coding RNA such as circRNAs and piwi-RNAs have been identified with diversified intracellular roles. Often categorized as a form of lncRNAs, circRNAs primarily originate from pre-mRNA and are generated through back-splicing, where the upstream 5′ terminus of an exon is spliced to the downstream 3′ terminus of an exon [366]. The resulting circular form of the circRNA product lacks the 5′cap and 3′ tail found in linearity, which promotes its resistance to degradation by RNase [366]. CircRNAs can functionally alter and regulate both protein and gene expression, resulting in a wide variety of effects on cellular and physiological processes important to proliferation, cell cycle, EMT, and cancer progression [367]. Furthermore, it has been well defined that through their capability to simultaneously bind numerous miRNAs, circRNAs can act to sponge and inhibit the activity of various miRNAs [366,367,368,369,370,371], and similar to what is observed with many lncRNAs, this can result in profound alterations to the biological and metabolic processes important to tumorigenesis [368]. Studies have revealed that various circRNAs may act as oncogenic promoters in breast cancer [372], NSCLC [373], melanoma [374], gastric cancer [375], gallbladder cancer [376], and CRC [377] models. Furthermore, some of these studies have revealed that circRNAs may enhance carcinogenesis through impacting glycolysis and PKM2 expression levels (**Table 5**). For instance, elevated levels of circMAT2B are associated with hepatocellular carcinoma (HCC) and predict poor prognosis. Additionally, the overexpression of circMAT2B under hypoxic conditions promotes glycolysis and malignancy through modulation of the miR-338-3p/PKM2 axis [282]. Similarly, Tian et al. demonstrated that circ-FOXM1 promotes melanoma cells’ proliferation and invasion through the regulation of glycolysis and PKM2 and Flotillin 2 levels. These effects were mediated through the regulation of miR-143-3p. Although the link between miR-143-3p and PKM2 is yet to be determined, the overexpression of miR-143-3p abolished lactate production and glycolysis in A2058 and A375 melanoma cells [374]. Likewise, the expression level of circ-NRIP1 in gastric cancer tissue and cells was shown to be upregulated. Conversely, the knockdown of circ-NRIP1 in AGS and HGC-27 GC cells resulted in reduced PKM2 expression levels, proliferation, glycolysis, migration, and apoptosis induction [375]. It was postulated that these antitumorigenic effects might have occurred, at least in part, through the competitive targeting of miR-186-5p. Parallel to these findings, circ-FOXP1 was shown to promote the Warburg effect through sponging miR-370 and subsequent regulation of PKLR in gallbladder cancer cells [376]. In addition, circ-FOXP1 silencing resulted in a partial reduction in PKM2 in vitro. In another elaborate CRC model, the role of exosome-delivered circular RNA hsa_circ_0005963 (ciRS-122) was investigated. The findings demonstrated that in both in vitro and in vivo models, ciRS-122 was delivered by exosome to chemosensitive cells and resultantly increased drug resistance and glycolysis by sponging miR-122 and upregulating PKM2 expression. In addition, mouse studies revealed that, through regulation of this ciRS-122/miR-122/PKM2 pathway, exosome-delivered small interfering (si)RNA targeting of ciRS-122 resulted in the reversion of chemoresistance and suppression of glycolysis [377]. These findings promote this innovative exosomal, nanoparticle delivery system as a potential therapeutic target with significant clinical implications. Collectively, the body of work presented thus far has revealed that through sponging miRNAs and regulating PKM2 expression, the various reported circRNAs may act as tumor promoters in numerous cancerous forms. However, the findings are still rather limited, and further exploration targeting the comprehensive effects of these circRNAs on the overall metabolic profile within normal and disease states is needed. Beyond circRNAs, other forms of non-coding RNA such as Piwi-interacting RNA (piRNA), a novel class of small non-coding RNAs [378], may be involved in mediating PKM2’s functions in health and disease. However, limited knowledge on this matter exists [379]. Nevertheless, the identified and emerging roles of piRNAs, suggesting their capacity to regulate various aspects of cancer, warrant future investigation.

## 5. Conclusions

PKM2 research remains integral to the pursuit of better cancer treatment options [27,29,96]. Understanding the functions and processes through which PKM2 affects the body and metabolism is intricate and complex. The effects on the genetic transcription of the specific PK isoform to the shift in quaternary structures between the active and inactive forms all play key functions in the overall metabolic and non-metabolic functions of PKM2 [30,60,71]. In order to promote translation, all factors that alter PKM2 function must be taken into consideration when designing therapeutic interventions targeting PKM2. Groundbreaking studies focused on new angles that push the boundaries have begun illuminating upon PKM2 roles previously undiscovered [25,91,154]. Moreover, it will be of the utmost importance to continue the exploration of PKM2, from cancer to the tissue- and hormone-specific effects [25,27,29,380]. Understanding PKM2 from all angles remains central to developing treatment options with less side effects. Continued exploration of the tissue-specific functions of PKM2 could lead to improved pharmacological intervention with more precise targeting and potentially fewer side effects. Furthermore, understanding the role of PKM2 within critical metabolic tissues, such as pancreas and liver, aids in clarifying the interactions involving PKM2 and cancer.

Beyond its structural and metabolic functions, the role of PKM2 and its interactions with miRNAs and lncRNAs have garnered increasing interest. Collectively, miRNAs and lncRNAs silencing or overexpression may possess profound therapeutic potential through the alleviation of off-target effects. The comprehensive finding suggests that through tissue-specific targeting of PKM2, these miRNAs and lncRNAs may, in theory, reduce the risks for side effects in both cancerous and non-cancerous disease states. Furthermore, recent biotechnological advancements regarding both traditional and innovative approaches may allow for improved targeting efficacy. One such avenue of interest revolves around the idea of generating either natural or synthetic nanoparticles [381]. In cancer research, the potential of nanoparticles is twofold, where they could be designed as nanoprobes to improve disease screening methods and enhance the therapeutic targeting of miRNAs [381]. Moreover, recent advancements into genome editing technology such as the CRISPR/Cas9 system also have identified potential for the targeting of miRNAs in various research models [382,383]. In addition, elevated serum levels of PKM2 have been observed in patients with various forms of cancer [384,385,386], and recent research has revealed that PKM2 serum levels may possess diagnostic potential as a biomarker for various diseases including lung cancer [387] and inflammatory bowel disease (IBD) [388]. The cure for cancer and the development of new and improved disease treatment and diagnostic options could lie in unravelling the mysteries of PKM2 [27,29]. This may occur through the implementation of these recent technological breakthroughs and further understanding their potential to reduce side effects. 

## Figures and Tables

**Table 1:** Roles of some PKM2-associated microRNAs in cancer.

miRNA	Research Model	Targeted Pathways	Effects on PKM2 Expression	Biological/Physiological Effects	Significance
miR-122	Hepatocellular Carcinoma (HCC)	Glycolysis Proliferation Apoptosis	Decreased PKM2 mRNA and protein levels (direct targeting)	Reduction of lactate production Increased oxygen consumption [287] Decreased glucose uptake and ATP production [288] Induction of apoptosis [288,296] Reduced tumor cell proliferation [296]	Targeting PKM2 through its negative regulator miR-122 may serve as a therapeutic approach to improve chemosensitivity [288] and survival among patients with HCC [296]
	Colon Cancer	Glycolysis	Decreased PKM2 protein levels through direct binding to PKM2 mRNA	Reduced glucose uptake and lactate production Reduced tumor cell viability and tumor volume Sensitization to 5-Fluorouracil therapy [280]	Targeting PKM2 through stabilization of miR-122 in colon cancer may enhance the effectiveness of chemotherapy in colon cancer
	Gallbladder Cancer	Proliferation Metastasis	Decreased PKM2 expression	Inhibits TGF-β-induced epithelium mesenchymal transformation Decreases proliferation and metastatic potential [312]	Targeting PKM2 through its negative regulator miR-122 may potentiate the effect on reducing cell invasion in gallbladder cancer
miR-372	HCC	β-catenin/Transcription Factor 4 Glycolysis	Increases the activity and expression of PKM2	Promoted liver cancer cell cycle progression via activation of the cell cycle complex CDK2-cyclin E-P21/Cip1/WAF1. Effects are mediated through miR372-YB-1-β-catenin-LEF/TCF4-PKM2-erbB-2 axis [316]	Targeting miR-372 may be of therapeutic value for the treatment of HCC
miR-374b		Glycolysis	Decreased PKM2 mRNA level (indirect regulation mediated through hnRNPA1 inhibition)	Sensitization to sorafenib therapy Reduces the number of developed colonies [297]	Enhancing miR-374b expression may promote the effectiveness of chemotherapy, halt tumor growth, and increase patient survival likelihood. These effects are mediated through the suppression of hnRNPA1 and its downstream effector PKM2
miR-199a		Glycolysis	Decreased PKM2 mRNA and protein levels (direct regulation)	Decreased glucose consumption and lactate production [317] Decreased cancer cell proliferation and survival Reduced tumor growth [112]	Using natural compounds that enhance miR-199a expression to suppress PKM2 may potentiate the effectiveness of HCC treatment [317]
miR-4417		Proliferation Apoptosis	No effect on PKM2 mRNA or protein levels	Decreased expression of TRIM35 Increased PKM2 Tyr-105 phosphorylation Increased proliferation Reduced apoptosis [308]	Targeting miR-4417 may serve as an adjuvant therapy to induce apoptosis and halt HCC growth mediated by inhibiting PKM2 Tyr-105 tyrosine phosphorylation
miR-199a-5p		HIF-1α	Decreased PKM2 mRNA level (indirectly by suppressing HIF-1α)	Decreased cell growth Reduced glucose uptake and lactate production [318]	Promoting miR-199a-5p expression may increase patient survival rate through suppressing the expression of HIF-1α and PKM2
miR-338-3p		Glycolysis Cell Migration	Decreased PKM2 mRNA level (direct binding leading to PKM2 mRNA degradation)	Sponging miR-338-3p mediated by circMAT2B resulted in increased glucose utilization, tumor expansion, and metastatic potential [282]	Enhancing miR-338-3p expression may reduce the progression of HCC and its metastatic potential
MiR-139-5p	Gallbladder Carcinoma	Glycolysis	Decreased PKM2 mRNA and protein levels (direct regulation)	Decreased glucose consumption, lactate production, and ATP availability Reduced cell proliferation and migration [283]	Overexpressing miR-139-5p may serve as a therapeutic approach to halt GBC progression and improve GBC patient outcomes
MiR-let-7a	Gastric Cancer (GC)	c-Myc hnRNPA1	Decreased PKM2 mRNA and protein levels (indirect regulation mediated through the reduction in c-Myc and hnRNPA1 expression)	Decreased cell proliferation and migration Reduced tumor size [313]	MiR-let-7a was reduced in GC tissues and enhancing its expression in GC might be capable of decreasing proliferation and metastatic potential
miR-124	Colorectal Cancer (CRC) Colorectal Adenoma (CRA)	PTBP1 DDX6	Increased PKM1 to PKM2 mRNAs ratio (indirect regulation mediated through the suppression of PTBP1) [314]	Increased oxidative stress, apoptosis, and autophagy [314] Modulation of the Warburg effect [314]	Expression of miR-124 is reduced in CRA and CRC. Its stabilization could suppress tumorigenesis through modulation of PKM1 to PKM2 ratio [314]
	Prostate Cancer	Proliferation	Decreased PKM2 protein levels	Reduced cell proliferation [319]	May suppress tumor growth through reducing the proliferation of prostate cancer cells
	Pancreatic Cancer (PDAC)	Autophagy Apoptosis	Decreased PKM2 protein levels (possibly mediated through the suppression of PTBP1)	Reduced autophagy and enhanced gemcitabine-induced apoptosis [304]	miR-124/PTBP1/PKM2 axis could play a pivotal role in gemcitabine resistance and miR-124 overexpression may have therapeutic potential
miR-124 miR-137 miR-340	CRC	Glycolysis Oxidative Phosphorylation	Increased PKM1 to PKM2 mRNAs ratio. Reduced PKM2 expression through indirect regulation mediated by PTBP1, hnRNAPA1, and hnRNAPA2	Decreased glycolysis rate and lactate production Increased oxygen consumption Inhibition of cell growth [289]	Enhancing the expression of any of the three miRNAs may increase survival rates and could exhibit antitumorigenic effects
miR-184	Clear-Cell Renal Cell Carcinoma (ccRCC)	Glycolysis	Decreased PKM2 mRNA and protein levels (direct binding to PKM2 mRNA)	Reduced glucose consumption and lactate production Reduced cell proliferation [284]	The negative correlation between miR-184 and PKM2 expression in human ccRCC samples suggests that targeting miR-184 may be of therapeutic value
miR-152	Breast Cancer (BC)	β-catenin Proliferation	Decreased PKM2 protein level (direct binding to PKM2 mRNA)	Reduced β-catenin Reduced cell proliferation and colony development [285]	Enhancing miR-152 may inhibit cancer cell proliferation and sensitize cancer cells to paclitaxel therapy through modulating the β-catenin pathway
miR-155		PIK3R1 FOXO3a c-Myc Glycolysis	miR-155 knockout caused a reduction in PKM2 mRNA and protein levels (indirect regulation mediated through the FOXO3a/c-Myc axis)	Increased glucose utilization [299]	miR-155 may promote tumorigenesis through upregulation of PKM2 and glucose metabolism. Reducing miR-155 levels could serve as a new therapeutic approach for breast cancer
miR-532-3p	Ovarian Cancer	Glycolysis DNMT3A	Decreased PKM2 protein level (an indirect regulation/unknown mechanism)	Decreased glucose consumption and lactate production [300]	Targeting miR-532-3p/PKM2 axis using glycosylated triterpenes such as 20(S)-Rg3 may prove a safe and effective therapeutic option and warrants further exploration
miR-29b		Glycolysis AKT2/3	Inhibition of miR-29b increased PKM2 mRNA and protein levels (indirect regulation mediated through the activation of AKT2/3)	Reduced glucose uptake and lactate production Reduced xenograft tumor formation [301]	miR-29b may disrupt glucose metabolism and suppress epithelial ovarian cancer growth by reducing AKT2-AKT3 and PKM2 levels
miR-338-3p		Glycolysis Proliferation	Decreased PKM2 mRNA and protein levels (direct binding to PKM2 mRNA)	Decreased ATP and lactate production Reduced cell proliferation [286]	Promoting miR-338-3p expression might be of therapeutic value due to its negative regulation of PKM2 in ovarian cancer cells
miR-145		Glycolysis Lactate Production Cell Growth	Decreased PKM2 protein levels (indirect mechanism mediated through upregulation of the c-Myc/miR133-b pathway)	Decreased glucose consumption and lactate production Reduced cell proliferation [320]	Promoting miR-145 expression might be a therapeutic strategy for the treatment of ovarian cancer through modulation of the Warburg effect
miR-148a miR-326	Thyroid Cancer	Cell Growth Apoptosis	Decreased PKM2 mRNA and protein levels (direct binding to PKM2 mRNA)	Reduced colony development and overall metastatic potential [290] miR-326 induced apoptosis in HeLa cells [305]	miR-148a and miR-326 may exhibit tumor-suppressive effects and reduce the metastatic potential of thyroid cancer cells [290] Identification of natural compounds that could regulate miR-326 such as resveratrol may serve as adjuvant therapy for thyroid cancer [305]
miR-326	Glioma	Metabolic Activity	Decreased PKM2 protein levels through direct binding to PKM2 mRNA	Reduced ATP levels [302]	miR-326 may exhibit tumor-suppressive effects through decreased PKM2 expression
miR-181b		Glucose Metabolism Colony Formation	Decreased PKM2 protein levels mediated by downregulation of Specificity protein 1 (SP1)	Reduced glycolysis, proliferation, and colony number [321]	Promoting miR-181b expression might be of therapeutic potential for glioblastoma multiforme
miR-214	Non-Small-Cell Lung Cancer (NSCLC)	Glycolysis PTEN Akt mTOR	Increased PKM2 mRNA and protein levels (indirect regulation mediated through PTEN and the AKT/mTOR pathway)	Increased glucose consumption and lactate production Increased cell proliferation [295]	miR-214 may act as an oncogene. miR-214 suppression could reduce PKM2 expression and cancer growth
miR-625-5p	Melanoma	Glycolysis Proliferation	Decreased PKM2 mRNA and protein levels (direct regulation)	Decreased glucose consumption, ATP and lactate production Reduced cell proliferation [293]	miR-625-5p may serve as a potential target to reduce cancer growth and glycolysis in human melanoma
miR-133a miR-133b	Tongue Squamous Cell Carcinoma (SCC)	Proliferation Apoptosis	Decreased PKM2 protein levels (direct regulation)	Decreased cell proliferation and induction of apoptosis [292]	miR133a and miR-133b may exhibit antitumorigenic potential and could serve as a potential therapeutic strategy for SCC
miR-133b miR-1	CRC	Proliferation Autophagy	Decreased PKM2 to PKM1 mRNA ratio and PKM2 protein expression levels (indirect regulation mediated through decreased PTBP1 levels)	Increased ROS Decreased lactate production Increased ATP level Induction of autophagy Reduced xenograft tumor volume [311]	Both miRNAs may have tumor-suppressive potential through their effects on PKM2 and glucose metabolism
miR-133b	NSCLC	Glycolysis	Decreased PKM2 protein levels (direct regulation)	Reduced glucose uptake and lactate production Sensitization of NSCLC to radiation [303]	May overcome radio-resistance and could decrease glycolysis
mIR-140-5p	Chronic Myeloid Leukemia	Glycolysis Proliferation Apoptosis	Decreased PKM2 protein levels (indirect regulation through modulation of sine oculis homeobox 1 (SIX1) gene)	Overexpression miR-140-5p in chronic myeloid leukemia cells inhibited cell proliferation and promoted cell apoptosis [322]	Targeting the miR-140-5p/SIX1 axis may be a potential therapeutic target for the treatment of chronic myeloid leukemia
miR-let-7a	Cervical Cancer	Proliferation Cell Migration Apoptosis	Decreased PKM2 protein level through direct binding to PKM2 mRNA	Decreased proliferation, metastasis, and tumor growth [291]	miR-let-7a may have tumor-suppressive potential against cervical cancer
miR-491-5p	Osteosarcoma	Proliferation	Decreased PKM2 mRNA and protein levels through direct binding to PKM2 mRNA	Decreased cell proliferation [315]	Targeting PKM2 through its negative regulator miR-491-5p by natural compounds such as Oviductus Ranae (OR) may reduce tumorigenesis [298]
	HCC	Glycolysis Proliferation Cell Migration	Decreased PKM2 mRNA level through direct binding to PKM2 mRNA	Decreased glucose consumption and lactate production Reduced cell proliferation and metastasis [298]	
miR-1294	Osteosarcoma	Proliferation Cell Migration Invasion Apoptosis	Decreased PKM2 mRNA and protein levels through direct binding to PKM2 mRNA	Reduced cell proliferation, invasion, and tumor growth, while inducing apoptosis [323]	Enhancing miR-1294 expression modulates PKM2 expression to suppress tumorigenesis, which could serve as a therapeutic strategy for osteosarcoma

**Table 2:** Roles of some PKM2-associated microRNAs in non-cancer disease.

miRNA	Tissue Expression/Distribution	Research Model	Target Genes/Pathways	Effects on PKM2 Expression	Biological/Physiological Effects	Significance
miR-99a	Liver	Human liver cells (cancerous and noncancerous)	Glycolysis mTOR HIF-1α	Inhibited insulin-induced PKM2 expression	Regulates insulin-induced mTOR and HIF-1α expression Regulates insulin-induced glucose uptake and lactate production [340]	Insulin may inhibit miR-99a to regulate PKM2 expression, which provides a novel biological mechanism of regulating glucose metabolism in the liver that warrants further investigation
miR-122		Chicken livers	Liver metabolism	Inhibition of miR-122 increased PKM2 mRNA level (direct regulation)	Inhibition of miR-122 decreased *FABP5* mRNA level [331]	miR-122 may regulate aspects of liver function and homeostasis including lipid and glucose metabolism
		Chicken livers and hepatocytes	Autophagy Oxidative stress	Decreased PKM2 protein levels	Overexpression of miR-122 promoted autophagy and ameliorated arsenic-induced liver damage via decreasing PKM2 levels [341]	Reducing PKM2 expression through promoting miR-122 may lead to novel treatment strategies against arsenic toxicity
miR-19a-3p	Brain	Astrocytes and neurons Cerebral ischemic injury	Glycolysis Apoptosis (Bax, Caspase 3) Adipor2)	Decreased PKM2 protein levels	Decreased glycolytic enzymes, glucose consumption, and lactate production Increased markers of apoptosis [335]	miR-19a-3p may play a role in the regulation of neural cell function and could serve as a potential target against cerebral ischemic injury
miR-143		Ischemic stroke Rat cortex neurons and astrocytes	Glycolysis HK2	Decreased PKM2 mRNA and protein levels	Decreased glucose uptake and lactate production [336]	miR-143 inhibition may have neuroprotective potential during ischemic brain injury (IBI)
	Heart	Endothelial cells	Glycolysis HK2 LDHA	Decreased PKM2 protein levels	Decreased ATP/ADP ratio, glucose consumption, and lactate production [337]	Overexpression of miR-143 may contribute to EC dysfunction through the suppression of glycolytic activity
miR-124		Pulmonary arterial hypertension (PAH)	Proliferation Glycolysis Mitochondrial reprogramming	Decreased PKM2 mRNA levels (indirect regulation mediated through PTBP1)	Decreased glycolysis and lactate production Decreased the proliferation of blood outgrowth endothelial cells (BOECs) from patients with heritable PAH Restored mitochondrial function [338]	Targeting the miR-124-PTBP1-PKM2 axis may be of therapeutic potential for the treatment of PAH
miR125a	Synovium	Psoriatic arthritis (PsA)	Glycolysis Migration Invasion	Suppression of PKM2 expression	miR-125a inhibition promoted EC tube formation, glycolysis, branching, migration, and invasion [342]	Potential therapeutic approach for the treatment of PsA

**Table 3:** Role of some PKM2/long non-coding RNA in cancer.

LncRNA	Research Model	Targeted Pathways	Mediator	Effects on PKM2 Expression	Biological/Physiological Effects	Significance
LncRNA MAFG-AS1	CRC	Proliferation Glycolysis Cell cycle Apoptosis	MiR-147b	A general trend of increased PKM2 mRNA levels	Increased proliferation, cell cycle progression, and cell invasion Decreased apoptosis [346]	MAFG-AS1 may sponge miR-147b to promote tumorigenesis and may play a role in CRC progression
LncRNA-FEZF1-AS1		Glycolysis	Direct binding to PKM2	Increased PKM2 stability and protein levels through direct binding	Increased PK activity and lactate production [349]	It is upregulated in CRC and correlated with PKM2 expression. The suppression of LncRNA-FEZF1-AS1 could exhibit therapeutic potential
LINC00689	Glioma	Glycolysis Proliferation Migration Invasion	miR-338-3p	Increased PKM2 protein levels	Silencing of LINC00689 suppressed glycolysis, cell proliferation, migration, and invasion [350]	The *LINC00689/miR-338-3p*/PKM2 axis may have oncogenic potential and modulating this axis may prove effective in glioma therapy
BCYRN1	NSCLC	Glycolysis Proliferation Invasion	miR-149	Increased PKM2 mRNA and protein levels	Overexpression of BCYRN1 induced glycolysis, cell proliferation, and invasion [34]	Inhibiting BCYRN1 or/and AC020978 might be a therapeutic target for NSCLC
AC020978		Glycolysis Proliferation		Increased PKM2 protein levels and stability through direct binding	AC020978 induced glycolysis and proliferation partially through enhancing PKM2 levels and its transactivation capability on HIF-1α [355]	
LINC01554	Liver Cancer	Glycolysis Cell growth Akt/mTOR		Decreased PKM2 protein levels (promotes ubiquitin-mediated degradation of PKM2)	Inhibited Akt/mTOR signaling to reduce glycolysis Reduced cell growth and colony formation [347]	LINC01554 may exhibit tumor suppressor activity and could serve as a prognostic biomarker
lncRNA MEG3		Epithelial to mesenchymal transition (EMT) Wound healing		Negatively correlates with PKM2 mRNA and protein levels	Arsenic trioxide inhibited HCC migration and EMT through promoting MEG3 and reducing PKM2 [66]	MEG3 may disrupt metastatic potential after arsenic trioxide treatment and may exhibit beneficial effects through suppressing PKM2 expression
		Cell growth	miR-122	Decreased PKM2 protein levels	Promoted the expression of miR-122 to downregulate PKM2 expression and its nuclear translocation Reduced β-catenin, cell proliferation, and tumor growth [89]	MEG3 may act as a tumor suppressor with potential in prognostic and therapeutic clinical application
lncRNA HULC		Autophagy PTEN AKT-PI3K-mTOR pathway		Increased PKM2 protein levels	Increased LC3II and Sirt1, resulting in elevated levels of autophagy Increased cell growth [354]	HULC may play a critical role in the progression of hepatocarcinogenesis
lncRNA-SOX2OT		Glycolysis Metastasis	miR-122-5p	Increased PKM2 protein levels	Increased glucose metabolism and PKM2 expression and exacerbates the metastatic potential of HCC [356]	Suppressing SOX2OT might be of therapeutic value to halt HCC metastasis
Linc-ROR	PDAC	Autophagy Apoptosis PTBP1	miR-124	Silencing ROR decreased PKM2 protein levels	Overexpression of ROR increased autophagy Silencing ROR enhanced gemcitabine-induced apoptosis [304]	Linc-ROR may sponge miR-124 (a negative regulator of PTBP1) to increase PKM2 levels and confer chemoresistance
LncRNA XLOC_006390	Cervical Cancer	Apoptosis Cell migration	miR-338-3p	Silencing XLOC_006390 decreased PKM2 mRNA and protein levels	XLOC_006390 may act as a negative regulator of miR-331-3p and miR-338-3p Promoted tumorigenesis and metastasis [353]	XLOC_006390 may hold value as a marker for cervical cancer with potential therapeutic targeting application
LncRNA *RPPH1*	Breast Cancer	Cell cycle Proliferation Colony formation	miR-122	Increased PKM2 mRNA levels	Promoted proliferation, colonization, and cell cycle progression [352]	*RPPH1* may act in an oncogenic manner and its suppression could possess therapeutic potential
	Alzheimer’s Disease	Apoptosis Endoplasmic reticulum (ER) stress	miR-326	Increased PKM2 protein level	Ameliorated amyloid β (Aβ) induced ER stress and apoptosis in neuroblastoma cells [357]	RPPH1 might be of therapeutic potential for future Alzheimer’s treatment options
TP53TG1	PDAC Gliomas/Brain Tumor Cells	KRAS Proliferation Colony formation Glycolysis Proliferation Migration	miR-96 ND	ND Decreased PKM2 mRNA levels under high and low glucose levels	Promoted cell proliferation and invasion and inhibited apoptotic cell death [358] Promoted cell proliferation and migration and inhibited apoptotic cell death [359]	TP53TG1 may inhibit the Warburg effect and halt cancer cell growth and metastasis
HOXB-AS3	CRC	Glycolysis PKM splicing miR-18a processing	hnRNP A1	Inhibition of hnRNP A1-dependent PKM splicing and PKM2 expression. Effects are mediated through the HOXB-AS3 peptide and not the LncRNA.	HOXB-AS3 peptide inhibits tumorigenesis and metabolic reprogramming in CRC cells [360]	HOXB-AS3 and SNHG6 might be viable targets for disrupting CRC metabolism and tumor growth
SNHG6		Glycolysis *PKM* splicing		Knockdown of SNHG6 decreased PKM2/M1 ratio	SNHG6 promotes hnRNPA1 to favor PKM2 expression and the subsequent increase in glycolysis [361]	
LncRNA H19	Ovarian Cancer	Glycolysis	miR-324-5p	Increased PKM2 protein level	Increased glucose consumption and lactate production [351]	20(S)-Rg3 may inhibit the Warburg effect and halt cancer cell growth through the H19/miR-324-5p/PKM2 axis
	NSCLC	EGFR AKT		Decreased PKM2 protein levels by promoting PKM2 ubiquitin mediated degradation	Suppression of H19 exacerbated erlotinib resistance (an EGFR inhibitor) by increasing PKM2, and the subsequent activation of AKT [362]	Targeting H19/PKM2 axis might be a viable option to overcome erlotinib resistance
LINC00504		Glycolysis Proliferation Apoptosis	miR-1244	Increased PKM2 mRNA and protein levels	LINC00504 knockdown suppressed proliferation and glycolysis, but induced apoptosis [363]	Inhibiting LINC00504 may exhibit therapeutic potential for ovarian cancer treatment
LincRNA-p21	Prostate Cancer	Glycolysis Proliferation Tumorigenesis		Decreased PKM2 mRNA and protein levels through indirect regulation mediated by PTEN/AKT/mTOR pathway	Reduced proliferation and tumorigenic potential Its downregulation increased glucose consumption, lactate production, and pyruvate levels [348]	LincRNA-p21 may be a viable target for disrupting PC metabolism and tumor growth

**Table 4:** Roles of some PKM2/long non-coding RNAs in non-cancer diseases.

LncRNA	Research Model	Targeted pathways	Mediator	Effects on PKM2 Expression	Biological/physiological Effects	Significance
LncRNA RPPH1	Alzheimer’s disease (AD)	Endoplasmic reticulum stress (ER stress) Apoptosis	miR-326	Not determined (a possible increase through blocking the role of MiR-326 in downregulating PKM2 expression)	RPPH1 overexpression decreased β25-35-induced apoptosis in SH-SY5Y cells through downregulating ER stress and modulating PKM2 activity [357]	RPPH1 acts as a molecular sponge for miR-326. LncRNA RPPH1 could attenuate ERS and apoptosis in neurodegenerative disorders
LncRNA-Malat1	Type 2 diabetes (T2D)	Glucose-stimulated insulin secretion Cell death	Ptbp1 (Increased)	Silencing malat1 leads to β-cell dysfunction and alterations in glucose-stimulated insulin secretion	LncRNA-Malat1 enhances Ptbp1 stability and PKM2 expression [365]	LncRNA-Malat1 could possibly serve as a potential therapeutic target for T2D through modulation of PKM2 activity

**Table 5:** Roles of CirRNAs associated with PKM2 in cancer.

CircRNA	Research Model	Targeted Pathways	Mediator	Effects on PKM2 Expression	Biological/Physiological Effects	Significance
Circ-MAT2B	Hepatocellular Carcinoma (HCC)	Glycolysis Cell proliferation Invasion	miR-338-3p	Overexpression of Circ-MAT2B increased protein and mRNA PKM2 levels	Circ-MAT2B overexpression increases HCC glucose utilization, tumor growth, and metastasis in vivo Circ-MAT2B overexpression promoted glycolysis, cell proliferation, migration, and invasion in vitro under hypoxia [282]	Circ-MAT2B is associated with and predicts poor prognosis of HCC Targeting circ-MAT2B may alleviate HCC burden
Circ-FOXM1	Melanoma	Proliferation Glycolysis Invasion Apoptosis	miR-143-3p	Silencing CircFOXM1 decreased PKM2 protein levels	Increased proliferation, glycolysis, motility, and decreased apoptosis [374]	Circ-FOXM1 may promote melanoma progression through the miR-143-3p/FLOT2 axis
Circ-NRIP1	Gastric Cancer	Proliferation Migration Glycolysis Apoptosis	miR-186-5p	Silencing of circ-NRIP1 decreased PKM2 protein levels	Circ-NRIP1 KD decreased proliferation, migration, and glycolysis but induced apoptosis [375]	Circ-NRIP1 promoted carcinogenesis and may have potential in prognostic and clinical application
Circ-FOXP1	Gallbladder Cancer	Proliferation Migration Invasion Apoptosis Glycolysis	miR-370	Silencing of circFOXP1 resulted in a partial reduction in PKM2 protein level	Promotes proliferation, invasion, migration and decreases apoptosis [376]	Circ-FOXP1 may sponge miR-370 and promote PKLR expression, enhancing tumor progression
Circ-RNA hsa_circ_0005963 (ciRS-122)	CRC	Glycolysis Apoptosis	miR-122	Exosome delivery of CiRS-122 upregulated PKM2 levels	CiRS-122 delivery may promote drug resistance and glycolysis, and exosome-delivered siRNA appeared to reverse the resistance to treatment [377]	CiRS-122 silencing may promote enhanced therapeutic effectiveness against oxaliplatin-resistant CRC

## Data Availability

Not applicable.

## Abbreviations

ACL	ATP-citrate lyase
ACR	albumin to creatinine ratio
AIF	apoptosis-inducing factor
AIM2	absent in melanoma 2
AKI	acute kidney injury
AKR1A1	aldo-keto reductase family 1, member A1
ALDOA	aldolase A
AMPK	adenosine monophosphate-activated protein kinase
Apaf-1	apoptotic protease activating factor-1
APC/C-Cdh1	anaphase-promoting complex/cyclosome-Cdh1
APC	adenomatous polyposis coli
ATF6	activating transcription factor 6
ATG	autophagy related protein
ATP	adenosine triphosphate
BAD	blc-2 associated agonist of cell death
BAPN	β-aminopropionitrile fumarate
BAX	BCL2-associated X protein
BC	breast cancer
Bcl2	b-cell lymphoma 2
BCL-2	b-cell lymphoma 2
bFGF	basic fibroblast growth factor
BH3	bcl-2 homology domain 3
BIM	Bcl-2-like protein 11
BMPR2	bone morphogenetic protein receptor 2
BUN	blood urea nitrogen
CAD	coronary artery disease
CARM	coactivator-associated arginine methyltransferase
Caspase	cysteine-aspartic acid protease
CCI	chronic constriction injury
ccRCC	clear-cell renal cell carcinoma
CD	Crohn’s disease
CD31	cluster of differentiation 31
CGNP	cerebellar granule neuron progenitor
CHOP	C/EBP homologous protein
CircRNA	circular RNA
COX	cyclooxygenase
CRC	colorectal cancer
DHAP	dihydroxyacetone phosphate
DN	diabetes nephropathy
DSS	dextrin sulfate sodium
EAE	autoimmune encephalomyelitis
eEF2K	eukaryotic elongation factor-2
EGF	epidermal growth factor
EGFR	epidermal growth factor
EIF2AK2	eukaryotic translation initiation factor 2 alpha kinase 2
EIF2α	eukaryotic translation initiation factor 2 alpha
EMT	epithelial-mesenchymal transition
eNOS	endothelial NO synthase
ER	endoplasmic reticulum
ERK	extracellular signal-regulated kinase
ESRD	end-stage renal disease
FADD	fas-associated protein with death domain
EXP5	exportin 5
FBP	fructose 1,6-bisphosphate
FGF21	fibroblast growth factor 21
FIH-1	asparaginyl hydroxylase factor inhibiting
GFR	glomerular filtration rate
GLUT1	glucose transporter 1
GM-CSF	granulocyte-macrophage colony-stimulating factor
GPR78	G protein-coupled receptor
GSK3β	glycogen synthase kinase 3 beta
H2O2	hydrogen peroxide
HDAC	histone deacetylase
HFD	high-fat diet
hGBM	human glioblastoma multiforme
HGC	human gastric cancer
HIF 1α	hypoxia-inducible factor 1-alpha
HK	hexokinase
HMGB	high mobility group box
hnRNPA	heterogenous nuclear ribonucleoprotein A
HSP	heat shock protein
IBD	inflammatory bowel disease
ICAM	intracellular adhesion molecule
IFNγ	interferon gamma
IGF	insulin growth factor
IL-1β	interleukin-1beta
IL-6	interleukin-6
IRE1α	inositol-requiring transmembrane kinase/endoribonuclease 1 alpha
JAK	Janus kinase
JNKs	c-Jun N-terminal kinases
LC3	microtubule-associated protein 1A/1B-light chain 3
LDHA	lactate dehydrogenase A
LMW-PTPs	low molecular weight protein tyrosine phosphatase
lncRNA	long non-coding RNA
LPS	lipopolysaccharides
MAPK	mitogen-activated protein kinase
MDM2	mouse double minute 2
MEF	mouse embryonic fibroblasts
miRNA	microRNA
MLC-2	myosin regulatory light chain 2
MS	multiple sclerosis
mTOR	mammalian target of rapamycin
NAC	n-acetyl-L-cysteine
NADH	nicotinamide adenine dinucleotide
NADPH	nicotinamide adenine dinucleotide phosphate
NF-κB	nuclear factor kappa light chain enhancer of activated B-cells
NK	natural killer cells
NLRC4	NLR family CARD domain containing 4
NLRP3	NOD-, LRR- and pyrin domain-containing protein 3
NLS	nuclear localization signal
NO	nitric oxide
NO	nitric oxide
OAA	oxaloacetate
NPC	nuclear pore complex
NSCLC	non-small-cell lung cancer
OGD	oxygen-glucose deprived
OXPHOS	oxidative phosphorylation
PARP	poly (ADP-ribose) polymerase
PDAC	pancreatic ductal adenocarcinoma
PDK	pyruvate dehydrogenase kinase
PD-L1	programmed death-1 (PD-1) ligand 1
PEP	phosphoenolpyruvate
PERK	protein kinase RNA-like endoplasmic reticulum kinase
PFK	phosphofructokinase
PHD	pyruvate dehydrogenase
PHD3	prolyl hydroxylase 3
PHGDH	phosphoglycerate dehydrogenase
PI3K	phosphoinositide 3-kinase
PIN1	peptidyl-prolyl cis-trans isomerase NIMA-interacting 1
PiRNA	piwi-interacting RNA
PK	pyruvate kinase
PKC	protein kinase C
PKM	pyruvate kinase M
Pol II	polymerase II
PSAT1	phosphoserine aminotransferase
PTB	polypyrimidine tract binding protein
PTEN	phosphatase and tensin homolog
PTP	protein-tyrosine phosphatase
PTP1B	protein tyrosine phosphatase 1B
RBM4	RNA-binding motif
RISC	RNA-induced silencing complex
ROS	reactive oxygen species
rPKM2	recombinant PKM2
SCC	squamous cell carcinoma
SIR	systematic inflammatory responses
SIRT	sirtuins
SOCS3	suppressor of cytokine signaling 3
SNAP	synaptosome-associated protein
SNP	single nucleotide polymorphism
SOD2	superoxide dismutase
SREBP	sterol regulatory element binding proteins
SRSF3	serine/arginine-rich splicing factor
STAT	signal transducer and activator of transcription
T2D	type 2 diabetes
TGIF2	TGFB-induced factor homeobox 2
TAAD	thoracic aortic aneurysm and dissection
Th17	T helper 17
TLR	toll-like receptor
TNF-α	tumor necrosis factor alpha
TRIM35	tripartite motif containing 35
UC	ulcerative colitis
UCP1	uncoupling protein 1
VDAC	voltage-dependent anion channel
VEGF	vascular endothelial growth factor
Wnt	wingless-related integration site
WT1	Wilms’ tumor 1
